# Bill morphology and species identity predict heat loss at high air temperatures among tidal marsh sparrows in saline environments

**DOI:** 10.1242/jeb.252510

**Published:** 2026-10-14

**Authors:** Mackenzie R. Roeder, Adrienne I. Kovach, Danielle L. Levesque, Brian J. Olsen

**Affiliations:** ^1^ School of Biology and Ecology, University of Maine, Orono, ME 04469, USA; ^2^ Department of Natural Resources and the Environment, University of New Hampshire, Durham, NH 03824, USA; ^3^ Maine Agricultural and Forest Experiment Station, University of Maine, Orono, ME 04469, USA

**Keywords:** Thermoregulation, Thermography, *Ammospiza maritima*, *Ammospiza caudacuta*, *Ammospiza nelsoni*

## Abstract

The interactions between thermal environments and morphology provide critical insights into how organisms adapt to ecological pressures. Leg and bill morphology in birds are commonly shaped by their thermal regime, with thermoregulatory use of the legs predicted by high temperature extremes and bill size limited by cold temperature extremes. This general pattern has a well-documented exception, however, where the bills of birds in saline habitats appear adapted to high rather than low temperatures. Here, we present a test of the thermoregulatory use of the bill for high temperatures in three *Ammospiza* sparrow taxa, which face dual challenges of heat stress and freshwater limitation during the breeding season while representing a gradient of specialization to tidal saltmarshes. We quantified heat dissipation across ambient temperatures (10–35°C) using thermal imaging and examined interspecific and intraspecific variation in bill-mediated heat loss and its relationship to bill size. We found that larger bills facilitated greater heat loss, with seaside sparrows, the most tidally adapted taxon, exhibiting the highest heat dissipation rates and thermal efficiency. Notably, seaside sparrows also displayed non-linear heat dissipation patterns consistent with physiological control, with rapid increases in heat loss up to a thermal breakpoint (21.2°C) before plateauing. In contrast, saltmarsh and Nelson's sparrows exhibited linear, passive heat dissipation patterns typical of other songbirds. These findings highlight the dual contributions of morphological and physiological adaptations to thermoregulation in a freshwater-limited ecosystem, report evidence of the most fine-tuned physiological control of bill vasculature yet observed in a passerine, and offer insights into the evolutionary pressures driving avian thermoregulatory strategies.

## Teaser

**Highlighted Article:** Among tidal marsh sparrows from freshwater-limited habitats, larger bills dissipate more heat in hot weather, though species differ in heat loss beyond bill size.

## INTRODUCTION

The dynamic interplay between organisms and their thermal environment is a focal point in physiological ecology and evolutionary biology. Temperature plays a role in shaping both the physiological processes and the evolutionary trajectories of species (Haynie, 2001), and accordingly organisms display a range of strategies to deal with fluctuating ambient temperatures (Haynie, 2001; Tattersall et al., 2012). Understanding these thermal adaptations provides insights into the fundamental principles of physiology and the relationships between environmental pressures and evolutionary responses.

Maintaining body temperature, or thermoregulation, involves varied combinations of biochemical, physiological and behavioral processes, as well as the modification of heat loads both internally and externally (Schmidt-Nielsen, 1997; Tattersall et al., 2012). In whole-body endotherms, such as mammals and birds (Grigg et al., 2022), thermoregulation manages the balance between metabolic heat production and heat exchange with the environment. Factors such as body size and shape, surface-to-volume relationships, body composition, vascularity and insulation effectiveness can all influence this exchange (Tattersall et al., 2016). Maintaining body temperature can be particularly challenging during temperature extremes, and endotherms employ various strategies to deal with high temperatures including facultative hyperthermia, evaporative cooling and modifications of thermal conductance (McKechnie and Wolf, 2019).

Birds, like other endotherms, have developed a diverse array of thermoregulatory mechanisms to help dissipate heat during high ambient temperatures. These include panting, microsite selection (e.g. seeking shade), ptilo-erection (adjusting feather position to regulate heat exchange) and bathing (Hafez, 1964; Tattersall et al., 2016). Recent evidence suggests that avian bills also play a crucial role in thermoregulation, either conserving or dissipating body heat based on environmental conditions (Chaves et al., 2023; Tattersall et al., 2009, 2016). Avian bills are highly vascularized and covered by a keratinized, water-impermeable sheath known as the rhamphotheca. In addition to their other functions, bills can act as effective thermal radiators (enlarged, poorly insulated but highly vascularized surfaces used in heat dissipation) which transfer heat from the body into the environment through increased blood flow to the bill's surface (Greenberg et al., 2012a,b; Hagan and Heath, 1980; Phillips and Sanborn, 1994; Tattersall et al., 2009, 2016, 2018). Further, bills have been shown to follow Allen's rule (Symonds and Tattersall, 2010), which describes global patterns in body shape in response to temperature (Allen, 1877). Because appendage size influences the relative surface area available for heat loss, smaller appendages reduce heat loss in cold conditions and larger appendages facilitate it in warmer climates (Allen, 1877).

Size and shape, however, are not the only determinants of the thermoregulatory capacity of appendages; physiological adaptations can also play a critical role. For example, bird legs, which also serve as important thermal radiators, do not follow the morphological patterns predicted by Allen's rule. Instead, they exhibit vascular mechanisms that regulate heat exchange differently at high and low ambient temperatures (McQueen et al., 2023; Tattersall et al., 2016). These physiological adaptations, such as counter-current vasculature or finer control over vasodilation and vasoconstriction, weaken the tradeoff between appendage size and heat loss, especially under cold temperatures, and allow a variety of sizes to thermoregulate efficiently across a wide range of temperatures. In species exposed to dynamic thermal environments, evolution may optimize plastic physiological controls instead of the size of static thermoregulatory radiators (McQueen et al., 2023; Tattersall et al., 2016).

Bird bills overall have less physiological control than legs, perhaps due to tradeoffs with the blood supply needs of the respiratory turbinates, the lack of bill-specific counter-current blood vessels or the proximity of the bill vasculature to the brain and sense organs (McQueen et al., 2023). As a result, size appears to be the primary axis of bill thermoregulatory adaptation and bill size exhibits larger latitudinal clines than legs do in many bird species (McQueen et al., 2023).

Across all measured birds, both legs and bills appear to be involved in thermoregulation, although their relative use and degree of thermoregulatory adaptation vary by environment. Smaller bills are common when there are low minimum environmental temperatures, while increased physiological control or thermoregulatory use of the legs is a common response to high maximum temperatures (Tattersall et al., 2016). Bill size of passerine birds in particular is better predicted by minimum winter temperatures than maximum summer temperatures (Subasinghe et al., 2024; Danner and Greenberg, 2015; Friedman et al., 2017; Fan et al., 2019). Thus, because of the more static temperature of bills relative to legs, thermoregulatory selection for avian bill size appears constrained by time periods when inflexible bill conduction is a liability – during either the coldest critical period (Symonds and Tattersall, 2010; Danner and Greenberg, 2015) or when temperatures are hotter than avian body temperature (Greenberg and Danner, 2012; Subasinghe et al., 2024).

There is, however, one large exception to this general pattern between bill size and temperature, one that suggests an additional important mechanism shaping avian thermoregulation. In North America, the bill size of passerines in saline wetlands correlates more strongly with high summer than with low winter temperatures (Greenberg et al., 2012a). This outlier suggests that water conservation may increase the adaptive advantage of the bill for thermoregulation. Passerines are one of the few avian orders that lack salt glands (Chiu et al., 2024), and thus their available thermoregulatory strategies face greater trade-offs with water conservation than those of other bird orders (Greenberg et al., 2012a). Among close taxonomic relatives, passerine bills are consistently larger in saline habitats compared with those in less saline areas, even under similar temperature regimes (Luther and Greenberg, 2011; Greenberg et al., 2012b).

The Greenberg–Tattersall corollary to Allen's rule describes this widespread increase in bill surface area for birds inhabiting freshwater-limited environments (Olsen et al., 2019). Because the bill rhampotheca is water impermeable, increased surface area allows for radiative heat loss without relying on evaporative cooling, a beneficial strategy in environments with scarce freshwater (Alhajeri et al., 2020; Cartar and Guy Morrison, 2005; Greenberg et al., 2012b; McQueen et al., 2023; Ryeland et al., 2017; Symonds and Tattersall, 2010). Greater reliance on the bill for thermoregulation under high maximum temperatures also appears in both passerine and non-passerine birds in humid environments, where the benefits of evaporative cooling are simply less available (Subasinghe et al., 2024). Thus, while we expect low temperatures to scale with the importance of the bill for thermoregulation overall, avian bill size should be shaped more strongly by high maximum temperatures whenever they are coupled with challenges for evaporative cooling – due to either the costs of water conservation or reduced efficacy of evaporative cooling under high humidity.

While the correlational evidence for the use of the bill as a thermal radiator in saline environments is strong, to our knowledge, there has been no direct, interspecific comparison of the thermoregulatory use of the bill of species where we should expect different degrees of bill adaptation to high temperatures (but see Greenberg et al., 2012b, for an intraspecific comparison, and Tattersall et al., 2018, for interspecific comparisons explained by morphology alone). As bill size experiences a suite of additional selection pressures (e.g. for foraging efficiency or song production), direct measurements of thermoregulation are necessary to account for other factors that could explain why bill size varies by habitat type. Here, we compared how three species inhabiting a saline environment, the Atlantic tidal saltmarsh of North America, differ in their thermoregulatory use of the bill under increasing temperatures. All three species are migratory, with similar wintering ranges and lower critical environmental temperatures. They differ, however, in their hypothesized time of association with the saline habitat and in the northern extent of their breeding range (and thus the maximum summer temperatures the greater population experiences). If the Greenberg–Tattersall corollary is explained by bill adaptation for thermoregulation in the context of increased need for water conservation, we expect that the species with the longest evolutionary association with the environment and the more southern breeding range would exhibit greater and more efficient use of the bill for heat loss.

Tidal salt marshes, the grassland ecotones between land and sea, impose harsh selective pressures on the few vertebrate species that inhabit them, including several endemic bird species (Greenberg, 2006). These environments are characterized by a scarcity of freshwater and high thermal stress during the summer breeding season. A unique subgroup of the grassland clade of Passerellid sparrows includes three members of the genus *Ammospiza* that breed in tidal marshes: the seaside sparrow, saltmarsh sparrow and Acadian subspecies of Nelson's sparrow. These species exhibit a suite of phenotypes associated with vertebrate life in tidal saltmarshes, collectively termed the saltmarsh syndrome (Grenier and Greenberg, 2006): increased kidney size, decreased brood or litter size, nesting strategies associated with tidal inundation, increased melanism, and larger bills in comparison with their closest inland relatives (Grenier and Greenberg, 2005). The degree of specialization appears to correlate with time since colonization, with species that have occupied tidal marshes longer exhibiting more pronounced adaptations and occupying lower elevations within marsh zonation (Grenier and Greenberg, 2005; Malamud-Roam et al., 2006; Maxwell et al., 2024).

The seaside sparrow (*Ammospiza maritima*) diverged approximately 3 MYA (Walsh et al., 2019; Walsh et al., 2021) from its common ancestor with the sharp-tailed sparrows (i.e. the saltmarsh sparrow, *Ammospiza caudacuta*, and Nelson's sparrow, *Ammospiza nelsoni*), has the broadest and southernmost breeding range (northern terminus at 43.0°N) and occupies low marsh more frequently than the other taxa (Quay et al., 1983; Greenlaw et al., 2022). It is the largest (in both body and bill size), darkest and only taxon with sexual dimorphism in bill morphology (Grenier and Greenberg, 2005). The saltmarsh sparrow, which diverged approximately 500,000 years ago from its common ancestor with Nelson's sparrow, breeds slightly further north (northern terminus at 44.0°N), occupies high marsh and is intermediate in body size and bill morphology (Greenlaw and Rising, 1994; Walsh et al., 2019). The Acadian subspecies of Nelson's sparrow (*Ammospiza nelsoni subvirgata*) has the shortest association with saltmarsh. The genetic dissimilarity between this subspecies and the two inland subspecies (*Ammospiza nelsoni altera* and *Ammospiza nelsoni nelsoni*) is consistent with tidal marsh colonization since the last glacial maximum 20,000 years ago (Walsh et al., 2017). Of the three *Ammospiza* species, this taxon has the broadest breeding habitat niche (Hodgman et al., 2002), the northernmost breeding range (northern terminus at 49.1°N), and is the smallest, lightest and least specialized morphologically (Greenlaw, 1993; Maxwell et al., 2024). All three taxa migrate to similar wintering ranges along the southeastern coast of the USA (Greenlaw et al., 2020, 2022), where mean daily minimum temperature normals (2006–2020) during the non-breeding period range from 1.5°C to 11.6°C (Palecki et al., 2021).

In this study, we used a portable field-laboratory setup to conduct thermography trials on the three *Ammospiza* sparrow species to quantify the thermophysiological response of each to increasing temperatures. We developed a protocol for rapid (less than 1 h) temperature exposure trials in the field to minimize stress on the birds. Our specific study objectives were to (1) quantify and compare the ability of the three tidal marsh sparrows to use their bills as thermal radiators; (2) assess the role of bill size variation in bill-mediated heat loss (e.g. do larger bills with greater surface area enhance heat exchange at a given ambient temperature?); and (3) test for evidence of interspecific differences in physiological control independent of bill size (e.g. differences in heat loss per unit of bill area).

We hypothesized that the bill functions as a thermal radiator in all three taxa, but that the extent of heat loss will reflect each species' degree of saltmarsh adaptation. Specifically, we expected seaside sparrows – with the largest bills, longest evolutionary history in tidal marshes and most southern breeding range – to exhibit the greatest bill-mediated heat loss, while saltmarsh and Nelson's sparrows would show less. Within species, individuals with larger bills should lose more heat as a result of increased surface area. Based on prior studies in other passerines (McQueen et al., 2023), we further hypothesized that bill surface temperature would remain consistently elevated above ambient temperature by a relatively fixed margin, indicating limited physiological control, unlike the dynamic vasomodulation observed in bird legs.

## MATERIALS AND METHODS

### Ethics statement

We conducted all fieldwork and bird handling procedures in accordance with ethical guidelines for the care and use of animals in research (Fair et al., 2010). This study was approved by the Institutional Animal Care and Use Committee (IACUC) at the University of Maine under protocol number A2019-04-02 and the University of New Hampshire under protocols 190401 and 220401. All birds were captured and processed following the guidelines set forth by the U.S. Geological Survey's Bird Banding Laboratory (BBL). Research was conducted under Federal Banding Permits 24045 and 23613, State Permits 2021-270 and 2022-659 (Maine), NH State Permits in 2020 and 2021, 1522002 (Connecticut), SC 2021-101 and SC2022-084 (New Jersey), and 2021-013 and 2022-016 (USFWS Special Use Permit: Edwin B. Forsythe NWR). Every effort was made to minimize stress to the birds, which were released immediately following the thermal trials at the site of capture, and no injuries or mortalities occurred during the course of this study.

### Data collection

#### Sampling and morphological measurements

We conducted thermography trials on seaside sparrows, *Ammospiza maritima* (Wilson 1811) (*n*=11), saltmarsh sparrows, *Ammospiza caudacuta* (Gmelin, JF, 1788) (*n*=20) and Nelson's sparrows, *Ammospiza nelsoni subrvirgata* (Dwight, J., 1887) (*n*=5), captured in 2021 and 2022 between June and August on their breeding grounds. All individuals were adult males in breeding condition based on plumage characteristics and the presence of a cloacal protuberance. We captured birds in mist nets at six sites along the northeastern Atlantic coast of the USA between 04:00 h and 13:00 h. These sites occurred within the northern half of the breeding range of the saltmarsh sparrow, the southern half of the breeding range of the Acadian Nelson's sparrow, and in the northern part of the breeding range on the Atlantic Coast for the seaside sparrow (Fig. 1; Table S1). We banded each bird with a uniquely numbered USGS aluminium band prior to processing. Importantly, some of the birds sampled at our New Hampshire site could have been hybrids, as hybridization between saltmarsh sparrows and Nelson's sparrows occurs in this region. For those individuals, we used genetic data from a RadSeq analysis (Kuras, 2024) to confirm species identification. For each individual, we collected morphological data for the calculation of volume and surface area for body regions of interest (body, legs and bill) with digital calipers to the nearest tenth of a millimeter. Leg measurements included tarsus length, tarsus depth at mid-point and tarsus width at mid-point; bill measurements included nalospi (the distance from the anterior edge of the nares to the bill tip), bill depth at the anterior edge of the nares, and bill width at the anterior edge of the nares; and body size was described using mass (grams) and wing chord length (mm).

**Fig. 1.**
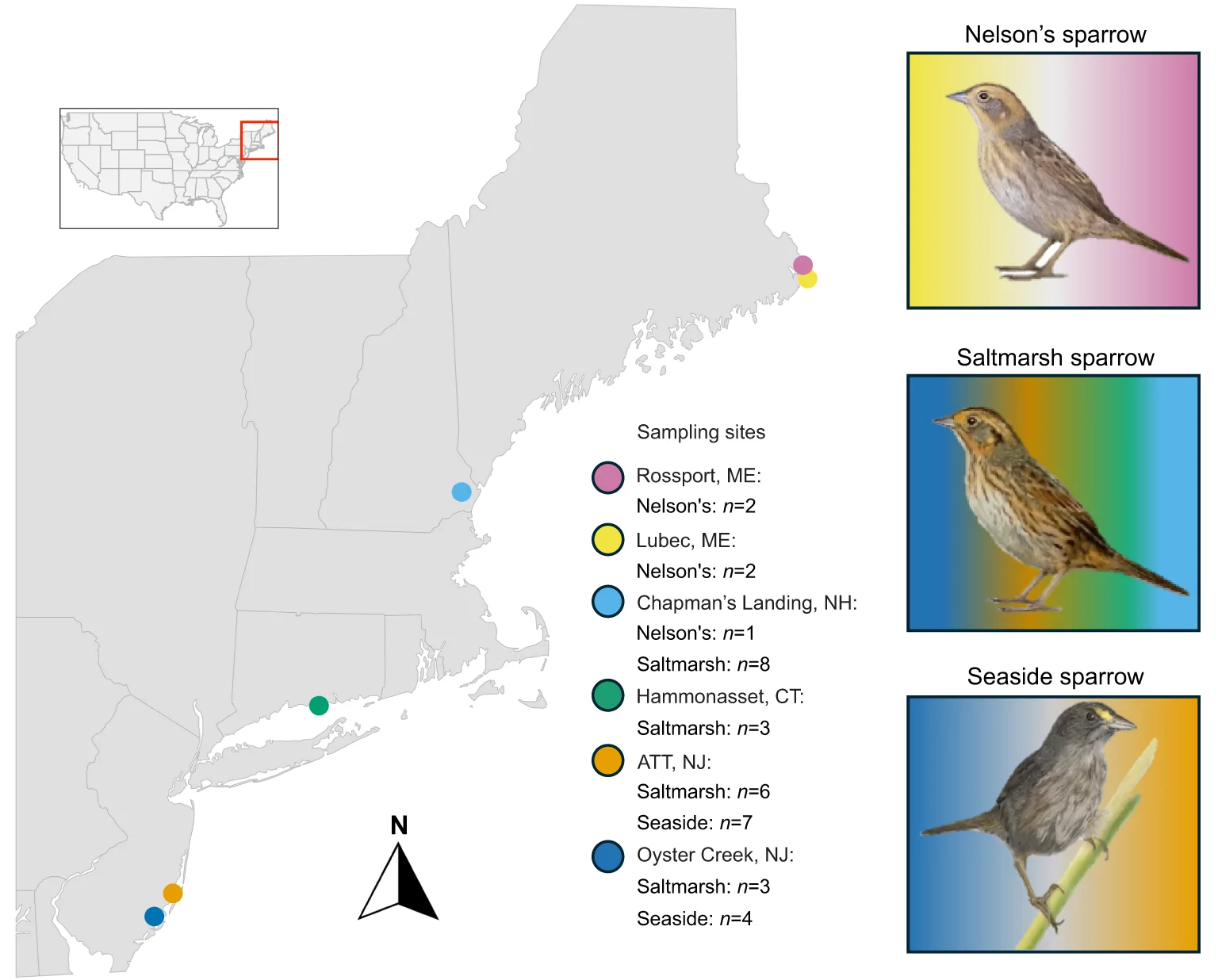
**Geographic distribution of thermography sampling sites and sample sizes across tidal marsh sparrow species.** Colored points indicate tidal marsh sampling locations from New Jersey to Maine (see Table S1). Numbers in the key indicate the exact number of individual birds sampled per species at each site (total number, seaside sparrows, *n*=11; saltmarsh sparrows, *n*=20; Nelson's sparrows, *n*=5). Thermography data were collected across two field seasons (2021 and 2022), and each individual was sampled once per capture event. Background gradients behind species illustrations indicate the sites at which each species was measured. ATT, American Telephone and Telegraph marsh. Illustrations were created by M.R.R. using SketchBook.

#### Morphological comparisons among species

To facilitate comparisons of morphological differences among the three tidal marsh sparrow species and establish a baseline for interpreting size-related variation in thermoregulatory traits, we conducted a series of ANOVAs on morphological traits, including body size and surface area measurements for body regions of thermoregulatory interest. These measurements allowed us to classify species-specific size differences and allowed for comparison to other studies on similarly sized birds with comparable bill dimensions. We used the morphological measurements to calculate key dimensions for regions of thermoregulatory interest for each individual and then grouped these traits by species for comparison. We calculated bill surface area of the distal bill (from the distal edge of the anterior nares to the bill tip) in mm^2^ by applying the formula for the lateral surface area of an approximately circular elliptical cone: ((width+depth)/4)×length×π (*sensu* Greenberg et al., 2012b). We calculated the surface area of the legs using the surface area formula for an elliptical cylinder (π×(2×((width/2)^2^+(depth/2)^2^)−0.5×(width−depth)^2^)^0.5^×length×2 (*sensu* Greenberg et al., 2012b). Body surface area was calculated following Walsberg and King (1978) and then multiplied by 100 to convert the units to mm^2^: 8.11×(mass^0.67^)×100. We also calculated the percentage of total surface area contributed by the bill, leg and body for each individual.

Summary statistics, including mean and standard deviation, were calculated for each species using the *get_summary_stats* function from the *rstatix* package in R (https://CRAN.R-project.org/package=rstatix). To assess morphological differences among species, we conducted a series of ANOVAs for the relevant body size and surface area traits, including wing chord, mass, tarsus length, distal bill surface area (mm^2^), tarsus surface area (mm^2^) and body surface area (mm^2^). For each of these traits, we tested for significant differences among species. Where assumptions of normality and homogeneity of variances were met, as assessed using Shapiro–Wilk and Levene's tests, respectively, we used standard ANOVA followed by Tukey's HSD *post hoc* tests for pairwise comparisons. When Levene's test indicated violations of the assumption of homogeneity of variances, we used Welch's ANOVA and conducted Games–Howell *post hoc* tests. Additionally, we compared the percentage contributions of each body region (bill, leg and body) to the total surface area by species. For these comparisons, we also used ANOVA with Tukey's HSD *post hoc* tests of significance.

### Thermal trials

To quantify the physiological responses of individual birds to a similar temperature ramp, we built a portable temperature-controlled chamber (hereafter thermal chamber, Fig. 2), which we used in the field. This approach allowed us to compare thermoregulation under identical temperature conditions, with only minimal, temporary impacts to breeding activities. The thermal chamber consisted of an insulated temperature-controlled assembly, incorporating an internal bird-housing chamber and a thermoelectric cooling unit that allowed us to create chamber temperatures well below and above the environmental temperatures at capture. The chamber was equipped with a CorDEX IW3000 infrared viewing window (Scarborough, UK) and a FLIR E-95 camera (42 deg lens, resolution 464×348; FLIR Systems, Wilsonville, OR, USA) to record thermal data. To limit stress-induced movements within the chamber, a cylindrical piece of light-blocking fabric around the viewing window on the cooler's exterior surface allowed for camera placement and bird observation while preventing external light from entering the chamber. Preliminary trouble-shooting of the design showed that birds held in darkness quickly ceased movement and stayed relatively still throughout the trial, while that was not the case when light entered the chamber. On either side of the bird-housing chamber within the cooler, we placed a professionally calibrated, ground-level, Tempo Disc Bluetooth-enabled thermometer, hygrometer and dew point logger (BlueMaestro, Montgomery, TX, USA) to record the chamber climatic conditions, with a reported temperature accuracy of ±0.1°C. For additional chamber design specifics see Supplementary Materials and Methods.

**Fig. 2.**
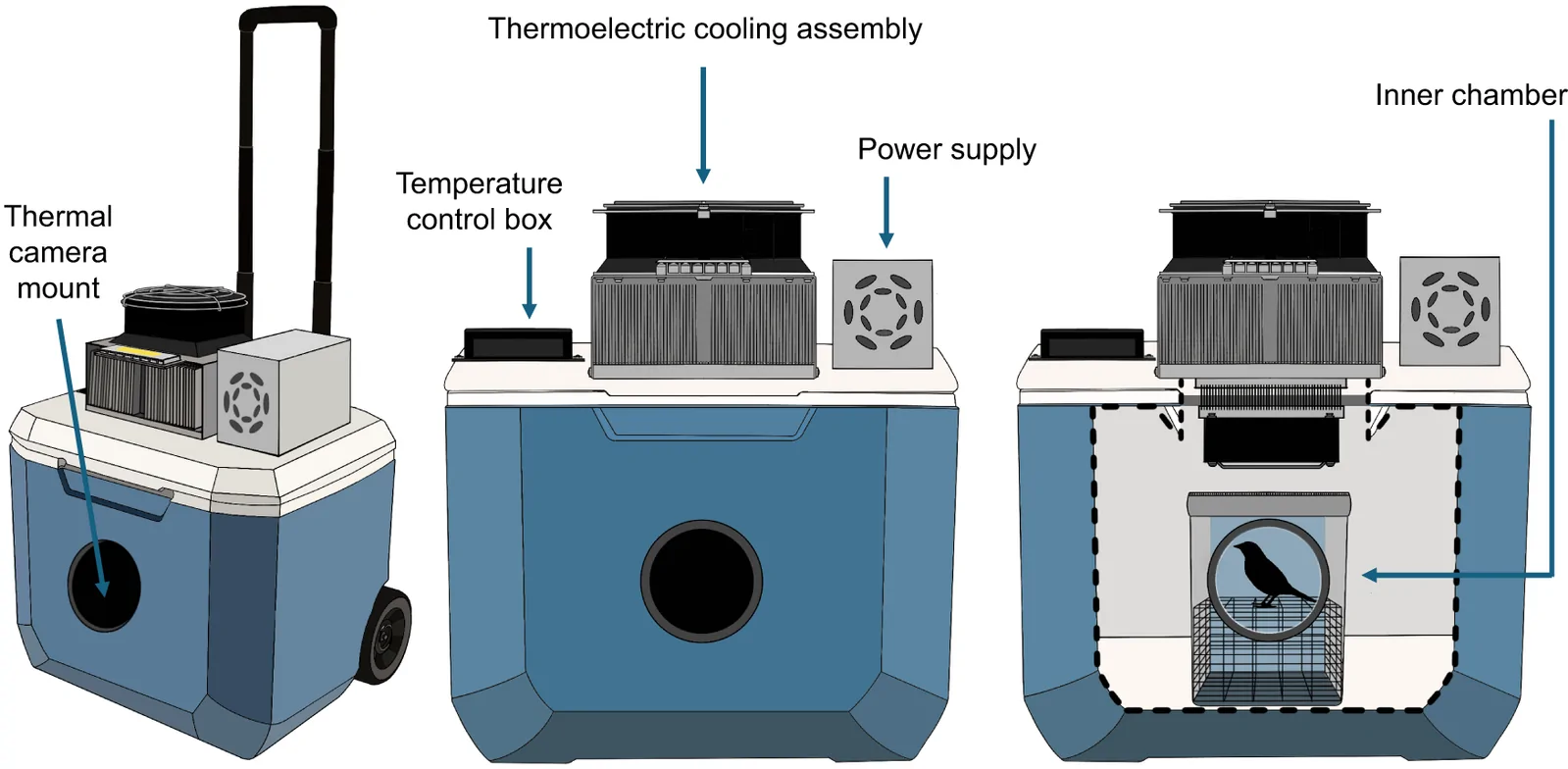
**Portable thermal chamber used for controlled thermography trials.** The system includes a thermoelectric cooling assembly connected to a temperature control unit and external power supply. Birds were placed in a ventilated inner compartment, and a mounted FLIR infrared camera captured thermal images through a viewing window. Illustration by M.R.R.

At the start of a mist-netting bout, the chamber was turned on to bring the internal temperature to 10°C. We transported individual birds, following capture and processing, to the marsh edge in a cloth bag before placing them into the thermal chamber, which was housed inside a parked vehicle. We then placed the bird into the bird-housing chamber inside the thermal chamber (see Fig. 2). We began recording with the thermal camera as soon as the lid of the thermal chamber was closed. Each trial exposed birds to a temperature range of 10°C to 35°C, representing typical mid-to-high summer temperatures encountered in their natural habitat. Trials began with a 10 min acclimation period at 10°C. This time period was adequate for all three species to become relatively calm and remain adequately still in the chamber to measure the temperature of the various body regions. Capture and handling in another passerellid sparrow (*Melospiza melodia*) reduced heat dissipation by the bill by 32.4%, but recovery to pre-capture baseline occurred in 2.3 min (Zuluaga and Danner, 2023). Our 10 min acclimation period was applied similarly to all individuals of all three species, although unanticipated species-specific stress responses significantly longer than that reported by Zuluaga and Danner (2023) could influence our results. Acclimation was followed by a series of 1 min ramp cycles and 5 min exposure periods at each subsequent temperature (15°C, 20°C, 25°C, 30°C and 35°C). Each trial lasted a total of 41 min. At the end of each trial, birds were immediately released back into the marsh, near their point of capture.

We collected additional temperature data for each trial to capture the birds' thermal environment, in case individual short-term environmental acclimation significantly impacted bird responses to our test chamber temperatures. When we captured a bird, we recorded ambient weather data including temperature, humidity and wind speed using a Kestrel 4000 Weather & Environmental Meter (Kestrel Instruments, Boothwyn, PA, USA), and recorded cloud cover using the US Weather Bureau sky condition codes. For the duration of the trial, we recorded data on the thermal conditions of the vehicle housing the thermal chamber (interior vehicle temperature and interior vehicle humidity) at each exposure temperature in the cycle. We used data from Weather Underground (https://www.wunderground.com/) to determine the mean temperature and humidity for the week prior to capture and trial.

We imported the .csq thermal video file from each trial into FLIR Research Studio (https://www.flir.com/products/flir-research-studio/) to isolate frames for analysis. Five frames were isolated from the last minute of each exposure temperature in the trial. Within each frame, landmarks were placed to isolate the following body regions of interest for measuring surface temperature: bill (the entire bill to the feather line), nalospi (the portion of the bill extending from the anterior edge of the nares to the bill tip), leg (the tarsus from the intertarsal joint to the distal joint where the toes articulate), and the body surface (the remainder of the bird with the exception of the bill, leg and external portion of the primaries that extend beyond the body). These regions were selected because of their involvement in thermoregulation and heat exchange, as indicated by prior studies (Greenberg et al., 2012b; Tattersall et al., 2009). We recorded the mean surface temperature (°C) for each region in each frame and averaged across all frames to obtain the mean region surface temperature per exposure temperature.

We calculated the average radiative and convective heat loss from each exchange surface: distal bill, leg and body (the external surface of the feathers across the body of the bird) following the methods of Tattersall et al. (2009) and Greenberg et al. (2012b). Total heat loss (*Q*) was calculated as the summation of radiative (*Q*_r_) and convective (*Q*_c_) heat transfer (Eqn 1), and whole-body heat loss as the summation of total heat loss across all heat exchange surfaces (Eqn 2). We used the following equations to calculate heat exchange:

total heat loss:

(1) $$Q=Q_{r}+Q_{c};$$

whole-body heat loss (where body regions of interest are numbered from 1 to *i*):

(2) $$Q_{t}=\;\overset{i}{\underset{1}{\sum }}Q_{ri}+\overset{i}{\underset{1}{\sum }}Q_{ci};$$

radiative heat loss:

(3) $$Q_{r}=\varepsilon \sigma \;A(T_{s}^{4}-T_{a}^{4}),$$

where ε is the emissivity of the surface exchange object and environment (assumed to be 0.97; Tattersall et al., 2009), σ is the Stefan–Boltzmann constant, *A* is the surface area (in m^2^), *T*_s_ is the surface temperature of the body region of interest and *T*_a_ is the ambient temperature (the temperature of the thermal chamber); and convective heat loss:

(4) $$Q_{c}=h_{c}A(T_{s}-T_{a}),$$

where *h*_c_ is the convective heat transfer coefficient for the body region, *A* is the surface area (m^2^), *T*_s_ is the surface temperature of the body region of interest and *T*_a_ is the ambient temperature (the temperature of the thermal chamber in K). The formula for the convective heat transfer coefficient is:

(5) $$h_{c}=\frac{\mathrm{Nu}\times k}{D},$$

where *k* is the thermal conductivity of air (W m^−1^ K^−1^) at each air temperature (*T*_a_), calculated as 0.0241+7.5907*e*^−6^×*T*_a_ (Tattersall, 2009), and *Nu* is the Nusselt number of each body region:

(6) $$\mathrm{Nu}=\mathrm{cR}e^{n}.$$

Here, *Re* is the Reynolds number, calculated as:

(7) Re=V×Dν,

where *V* is air velocity (m s^−1^), *D* is the critical dimension [in m; length for the legs, depth for the bill and $\sqrt[{3}]{\mathrm{volume}}$ for the body, where volume (mass/density) was calculated using the estimated density ρ of a sparrow (0.913 g cm^−3^; Seamans et al., 1995)] and ν is kinematic viscosity of air at each ambient temperature (*T*_a_):

(8) $$\nu =-1.088e^{-5}+8.85e^{-8}\times T_{a}.$$

We treated the bill as a nearly circular elliptical cone, the legs as a cylinder and the body as a sphere. The values of the constants *c* and *n* in the calculation of *Nu* (Eqn 6) are determined by the value of *Re*; when, for non-spherical regions, *Re*≥4000, *c*=0.174 and *n*=0.618; when *Re*<4000, *c*=0.615 and *n*=0.466 (Tattersall et al., 2009). For spherical regions (the body) values of *c* and *n* were 0.34 and 0.60, respectively (Mitchell, 1976). All estimates of *Re* for the bill and legs were <4000; thus, for the birds in our study and for the body regions measured, we used the following values of *c* and *n*: bill and legs, *c*=0.615, *n*=0.466; body, *c*=0.34, *n*=0.60.

To account for potential confounding factors, we tested bird activity rate within the chamber, local climatic acclimation (i.e. trial date and local temperature for both the hour and the week prior to capture) and variation in relative humidity. Models of bill surface temperature with any combination of the local acclimation variables performed worse (ΔAICc>2.0; Table S3) than a null model without them, and relative humidity within the chamber varied consistently with programmed changes in ambient temperature (Table S4), showing no independent effect on variables of interest (see Supplementary Materials and Methods for complete details). As a result, only activity rate was retained as a fixed effect in downstream analyses because of significant individual variation, while local temperature acclimation and relative humidity were excluded.

### Tidal marsh sparrow bills as thermal radiators

To investigate the role of the bill as a thermal radiator in tidal marsh sparrows, first, we aimed to assess whether the surface temperature of the bill changed in relation to ambient temperature. Next, we examined whether heat loss from the bill varied with ambient temperature, and finally, we explored whether the proportion of total heat loss attributable to the bill changed with ambient temperature. For each of these three questions, we also investigated whether these patterns and relationships were consistent among species.

#### Does bill surface temperature increase with ambient temperature?

To test whether the surface temperature of the bill changed in relation to ambient temperature, we used an information-theoretic approach (AICc) to compare multiple linear mixed models (LMMs) using the *lme4* package (Bates et al., 2015) and the AICcmodavg package (https://cran.r-project.org/web/packages/AICcmodavg/index.html) to identify the best set of predictors of bill surface temperature. Candidate models were fitted using maximum likelihood for model comparison, and the best-supported model was subsequently refitted using restricted maximum likelihood for parameter estimation. Our null model included individual identity as a random effect (to control for multiple measurements over each trial) and individual activity level as a fixed effect (to control for linear relationships between bird activity and body temperature). We then considered the performance of this null model relative to models containing additional fixed effects of interest – species identity and linear, quadratic and cubic versions of ambient temperature (*T*_a_, *T*_a_^2^ and *T*_a_^3^) – as well as all of the two-way interactions between species identity and ambient temperature (both linear and curvilinear). Models with ΔAICc<2.0 were considered equivalent. We used a model selection approach instead of a more traditional hypothesis-testing framework because *P*-value calculations are anti-conservative in the *lme4* package, due to the difficulty of estimating degrees of freedom accurately in a mixed model framework. Information-theoretic approaches can overcome this limitation, provided the random structure is shared among all models, as was the case here (Bolker et al., 2009). We standardized (centered and scaled) all continuous variables and defined *T*_a_ as the mean value measured in the thermal chamber over the final exposure minute for each exposure temperature within the trial. We compared the performance of linear (*T*_a_) versus curvilinear (*T*_a_^2^ and *T*_a_^3^) relationships with ambient temperature to account for potential non-linearities with bill surface temperature. For models containing interactions, we used the *emmeans* package (https://cran.r-project.org/web/packages/emmeans/index.html) to estimate fitted means and Tukey-adjusted pairwise species contrasts at each experimental temperature (10–35°C) and to estimate species-specific trends where appropriate.

We performed four additional, similarly structured, model selection exercises with different dependent variables. The first two included models of the surface temperature of either the body or the legs to place bill thermoregulation in the context of other heat-exchange surfaces. The third modeled the total heat lost (in watts) from the bill over each trial temperature, and the last modeled the proportion of heat lost from the bill relative to that lost from the bill, body and legs combined.

### The role of bill size in bill-mediated heat loss

To explore how bill size influences the bill's role as a thermal radiator, we examined two key aspects. First, we investigated whether the size of a bird's bill is simply a product of body allometry both within and between species. Second, we investigated whether larger-billed birds lose more heat than would be predicted simply from their larger surface area for heat exchange. We assumed that any residual relationship between heat loss and species, after controlling for bill size, would suggest the presence of other thermoregulatory factors, such as physiological control.

#### Allometry – is bill size a product of body size?

To determine whether bill size differences among tidal marsh sparrows are primarily a function of body size (i.e. allometry), we conducted a principal components analysis (PCA) followed by a principal component regression (PCR). These analyses allowed us to assess whether bill size scales with overall body size across the three species. First, we calculated the natural logarithms of bill surface area (surface area of the distal portion of the bill in square millimeters) as well as three body-size parameters: mass, tarsus length and wing chord. This transformation helped ensure that the relationships among these variables were linear and suitable for regression analysis. We applied a PCA to the body size measures (mass, tarsus length and wing chord) using the *prcomp* function in R (https://www.r-project.org/). The PCA was conducted with centering and scaling to standardize the variables. Principal component scores (PC1) were extracted and used as a proxy for overall body size, as PC1 represented the majority of variance in body size across individuals, and all individual loadings were positively correlated with PC1.

To examine the relationship between bill size and body size, we then performed a PCR using the natural log of bill surface area as the response variable and PC1 (body size index) as the predictor. We also included species and the interaction between PC1 and species to compare allometric relationships across and within seaside sparrows, saltmarsh sparrows and Nelson's sparrows.

#### Do larger-billed birds lose more heat just because they have a larger radiator?

To assess whether larger-billed birds lose more heat simply because of the larger bill surface area, we again used an information-theoretic approach and a set of candidate LMMs to investigate the relationship between heat loss from the bill (in watts) and bill surface area (mm^2^). The null model again had a random effect for individual identity and a fixed effect for individual activity levels within the chamber. Candidate fixed effects included bill surface area, linear and curvilinear terms for chamber ambient temperature (*T*_a_, *T*_a_^2^, *T*_a_^3^), species identity and their two-way interactions.

### Physiological control over bill-mediated heat exchange

To look for evidence of physiological control over rates of heat dissipation from the bills of tidal marsh sparrows, we investigated whether the observed patterns of heat loss could be explained solely by morphological factors (e.g. bill size) or whether there is evidence for additional physiological control. To assess this, we conducted two complementary analyses. First, we assessed thermal efficiency (the amount of heat lost per unit bill surface area) to test whether efficiency varied among species and across ambient temperatures, while controlling for morphological differences in bill size. Second, we explored the potential physiological regulation of bill surface temperature by identifying breakpoints in its relationship (slope) with ambient temperature, which would indicate active switch points for the regulation of heat loss.

#### Thermal efficiency of heat loss from the bill

We calculated thermal efficiency by dividing total heat loss from the bill by the bill's surface area, normalizing heat dissipation per unit surface area. This metric allowed us to assess whether species differed in their thermal efficiency independent of bill size. We conducted a model selection exercise with thermal efficiency as the response variable and an otherwise identical structure to those described above. This analysis enabled us to test whether heat dissipation efficiency is driven primarily by species identity, temperature or their interaction, shedding light on potential physiological mechanisms underlying bill-mediated heat loss.

#### Testing for physiological control of bill-mediated heat loss

To investigate evidence of physiological control, we employed breakpoint regression analyses using segmented LMMs. Breakpoint regression allows us to detect whether the slope of the relationship between bill surface temperature and *T*_a_ changes at a certain threshold (i.e. a breakpoint). We fitted segmented LMMs using the *segmented.lme* function in R. The models included a random intercept for individual identity. Bill surface temperature was the dependent variable and *T*_a_ was the independent variable. Each species was modeled individually to assess species-specific differences in physiological control. The presence of a breakpoint was tested by comparing models with and without a breakpoint using AICc. Additionally, we assessed whether the slope of the relationship between bill surface temperature and *T*_a_ changed before and after the breakpoint. Because *segmented.lme* only allows for the estimation of a single breakpoint, we additionally fitted ordinary segmented regressions allowing up to two breakpoints to explore whether the relationship contained evidence of multiple potential changes in slope. As earlier analyses suggested non-linearity in leg surface temperature similar to that shown for other species by McQueen et al. (2023), we conducted the same breakpoint analyses for leg surface temperature as well.

## RESULTS

### Morphological comparison among species

Morphological comparisons revealed significant size differences among Nelson's sparrows, saltmarsh sparrows and seaside sparrows, with seaside sparrows consistently having the largest values across all traits, followed by saltmarsh sparrows, with Nelson's sparrows as the smallest (Table S2). Seaside sparrows had the longest wing chord, highest body mass and largest overall body surface area. Of particular relevance, seaside sparrows exhibited the largest distal bill surface area (mean±95% confidence interval, CI=92.4±4.3 mm^2^), which was significantly greater than that of saltmarsh sparrows (66.8±3.8 mm^2^) and Nelson's sparrows (63.6±3.5 mm^2^). The proportion of total body surface area made up by bill surface area was also significantly larger for seaside sparrows (2.3±0.1%) compared with saltmarsh sparrows (1.93±0.1%) and Nelson's sparrows (1.98±0.2%).

### Tidal marsh sparrow bills as thermal radiators

#### Does bill surface temperature increase with ambient temperature?

The best-supported model assessing the relationship between bill surface temperature and ambient temperature (*T*_a_) included interactions between species and both the linear and quadratic terms for *T*_a_. This model revealed a strong positive relationship between *T*_a_ and bill surface temperature across all species (Table 1; Table S5). At the mean experimental *T*_a_ (22.6°C), seaside sparrows had the highest bill surface temperature, 2.0±0.6°C higher than that of Nelson's sparrows, while saltmarsh sparrows had bill surface temperatures 0.7±0.5°C lower than that of Nelson's sparrows. Seaside sparrows also exhibited a significant non-linear response to *T*_a_, with bill surface temperature increasing rapidly at lower to moderate *T*_a_ and plateauing at higher temperatures (quadratic trend=−0.59, 95% CI: −0.86 to −0.32). In contrast, there was no significant evidence of non-linearity for Nelson's (quadratic trend=0.04, 95% CI: −0.31 to 0.38) or saltmarsh sparrows (quadratic trend=0.16, 95% CI: −0.02 to 0.34; Fig. 3A). The second-ranked model (ΔAICc=5.75) included an additional cubic interaction between species and *T*_a_ and supported similar conclusions, with *T*_a_ positively related to bill surface temperature and a non-linear response in seaside sparrows.

**Fig. 3.**
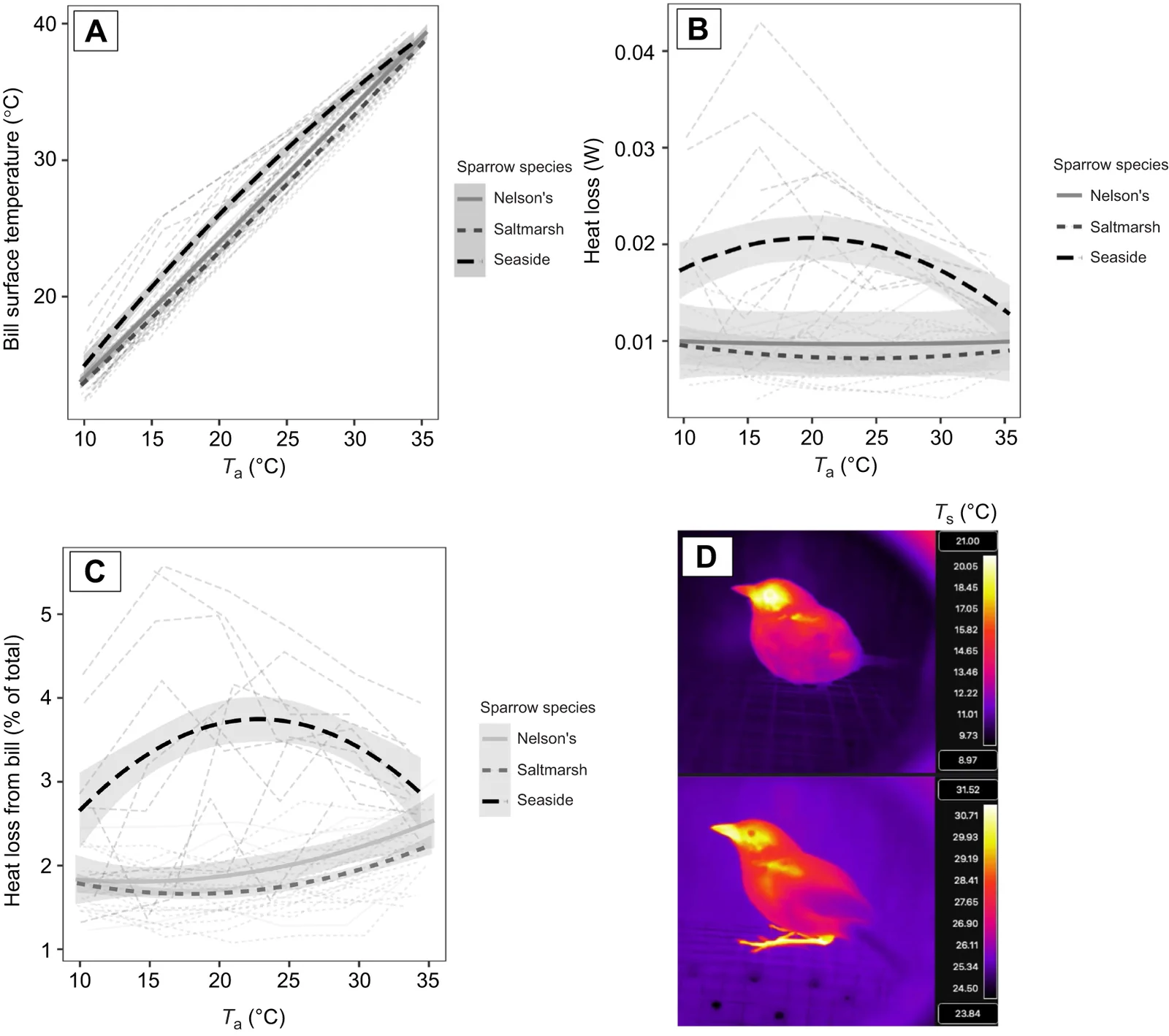
**The avian bill functions as a thermal radiator, with species differences in heat loss across increasing temperatures.** (A) Relationship between bill surface temperature and ambient temperature (*T*_a_) for seaside sparrows (*n*=11 individuals), saltmarsh sparrows (*n*=20) and Nelson's sparrows (*n*=5). Thin, semi-transparent lines represent observed repeated measurements from individual birds across increasing ambient temperature; thick lines show species-level predictions from the linear mixed-effects model; shading indicates the 95% confidence interval (CI). (B) Relationship between bill heat loss and ambient temperature for each species. (C) Relationship between heat loss from the bill expressed as a percentage of total body heat loss and ambient temperature for each species. (D) Representative thermal images of a seaside sparrow under cool (∼10°C) and warm (∼30°C) conditions. Color scales indicate surface temperature (*T*_s_).

**Table 1.** Model comparison for linear mixed models describing bill surface temperature responses to ambient temperature in tidal marsh sparrows

Model	*K*	AICc	ΔAICc	AICc wt
Species×*T*_a_+Species×*T*_a_^2^	12	634.21	0.00	0.94
Species×*T*_a_+Species×*T*_a_^2^+Species×*T*_a_^3^	15	639.96	5.75	0.05
Null model	7	1471.82	837.61	0.00

Models within ΔAICc<10 and the null model are shown; the full model set is provided in Table S5.

#### Body and leg surface temperatures

Across all three species, body surface temperature closely tracked ambient temperature. The top-ranked model for body surface temperature included only *T*_a_ as a predictor, although models including non-linear terms or additive effects of species and *T*_a_ were within 2ΔAICc (Table S9, Fig. S2). Predicted body surface temperature (*T*_s_) increased linearly with *T*_a_ and remained closely parallel with the 1:1 line (*T*_s_=*T*_a_) across the full experimental range (Figs S1 and S2). In contrast, leg surface temperature showed evidence of non-linear responses to ambient temperature. The best-supported model for leg surface temperature included linear, quadratic and cubic terms of *T*_a_, whereas models including species or interaction terms received considerably less support (Table S10). When pooled across species, leg surface temperature rose steeply with *T*_a_ at intermediate temperatures but showed shallower slopes at the lowest and highest chamber temperatures (Fig. S3). This pattern is consistent with stronger physiological control of heat exchange at the legs than at the feathered body surface and broadly aligns with previous work highlighting avian legs as key sites of thermoregulatory heat exchange. Because leg and body patterns largely matched expectations from previous work, our subsequent analyses focus predominantly on bill dynamics and bill-mediated heat loss.

#### Does heat loss from the bill vary with ambient temperature?

Consistent with the results for bill surface temperature, the best-supported model for heat loss from the bill included interactions between species and both the linear and quadratic terms for *T*_a_ (Table 2; Table S6). Seaside sparrows showed a non-linear response of bill-mediated heat loss to *T*_a_, whereas temperature-dependent changes were less pronounced for Nelson's and saltmarsh sparrows. Seaside sparrows exhibited a significantly greater amount of heat loss through their bills than the other two taxa from 10 to 30°C, with the greatest differences at 20°C (0.011 W more than Nelson's sparrows, 95% CI: 0.006–0.016 W; and 0.012 W more than saltmarsh sparrows, 95% CI: 0.009–0.016 W). At 35°C, these differences were reduced to 0.003 W more than Nelson's sparrows (95% CI: −0.003–0.009 W) and 0.004 W more than saltmarsh sparrows (95% CI: −0.0001–0.008 W) and were no longer statistically significant. The difference between saltmarsh and Nelson's sparrows was small and not statistically significant across the experimental temperature range (Fig. 3B). The second-ranked model included an additional cubic interaction but received less support (ΔAICc=6.2); all remaining models had ΔAICc values >15.3. Across the range of exposure temperatures, seaside sparrows lost between 46.7–148.2% and 32.6–113.2% more heat from their bills than saltmarsh and Nelson's sparrows, respectively. The best-supported model predicting the amount of heat lost via the bill (as a percentage of total heat lost) included interactions between species and both the linear and quadratic terms for *T*_a_ (Fig. 3C; Table S7). Seaside sparrows lost between 1.3% and 5.6% of total body heat via their bills over the course of the experiment; saltmarsh sparrows lost between 1.1% and 2.8%, and Nelson's sparrows lost between 1.2% and 3.0%. Seaside sparrows showed a non-linear response of bill-mediated heat loss as a percentage of total heat loss to *T*_a_; temperature-dependent changes were, again, less pronounced for Nelson's and saltmarsh sparrows. Seaside sparrows exhibited a significantly larger proportion of heat loss through their bills compared with the other two taxa from 10 to 30°C, with the greatest differences at 20°C. At 20°C, seaside sparrows lost 3.65% of total body heat through their bills (95% CI: 3.32–3.97%) compared with 1.90% for Nelson's sparrows (95% CI: 1.44–2.36%) and 1.67% for saltmarsh sparrows (95% CI: 1.43–1.90%). At 35°C, the differences among species were no longer statistically significant: seaside sparrows lost 2.73% of total body heat through their bills (95% CI: 2.33–3.14%), compared with 2.50% for Nelson's sparrows (95% CI: 1.94–3.05%) and 2.23% for saltmarsh sparrows (95% CI: 1.95–2.51%). The difference between Nelson's and saltmarsh sparrows was small and not statistically significant across the experimental temperature range. The second-ranked model included an additional cubic interaction but had less support (ΔAICc=5.9); all remaining models had ΔAICc values >32.5. Across the range of exposure temperatures, seaside sparrows lost between 22.6–118.7% and 9.5–92.0% more heat, as a percentage of total heat lost, through their bills than saltmarsh and Nelson's sparrows, respectively.

**Table 2.** Model comparison for linear mixed models describing heat loss from the bill surface across tidal marsh sparrow species

Model	*K*	AICc	ΔAICc	AICc wt
Species×*T*_a_+Species×*T*_a_^2^	12	−1759.40	0.00	0.95
Species×*T*_a_+Species×*T*_a_^2^+Species×*T*_a_^3^	15	−1753.25	6.15	0.04
Null model	4	−1709.43	49.97	0.00

Models within ΔAICc<10 and the null model are shown; the full model set is provided in Table S6.

### The role of bill size in bill-mediated heat loss

#### Allometry – is bill size a product of body size?

Although both seaside sparrows (β±s.e.=0.44±0.07, 95% CI: 0.30 to 0.57) and saltmarsh sparrows (β±s.e.=0.13±0.05, 95% CI: 0.04 to 0.22) had significantly larger bills than Nelson's sparrows (Table S2), the first principal component for body size (PC1) was not a strong predictor of bill surface area when considering all three species together (β±s.e.=−0.04±0.02, 95% CI: −0.08 to 0.001), suggesting that interspecific differences in bill size were not simply a byproduct of body size variation. However, when analyzed separately by species, the relationship between body size and bill surface area was significant for saltmarsh sparrows (β±s.e.=0.07±0.04, 95% CI: 0.004 to 0.143), indicating that within this species, larger individuals tended to have larger bills (Fig. 4A). In contrast, the relationship between body size and bill size was weaker and non-significant for Nelson's sparrows (β±s.e.=−0.04±0.02, 95% CI: −0.08 to 0.001). The slope for seaside sparrows (β±s.e.=0.04±0.03, 95% CI: −0.03 to 0.10) did not significantly differ from that of Nelson's sparrows, suggesting body size played a lesser role in predicting bill surface area for these two taxa.

**Fig. 4.**
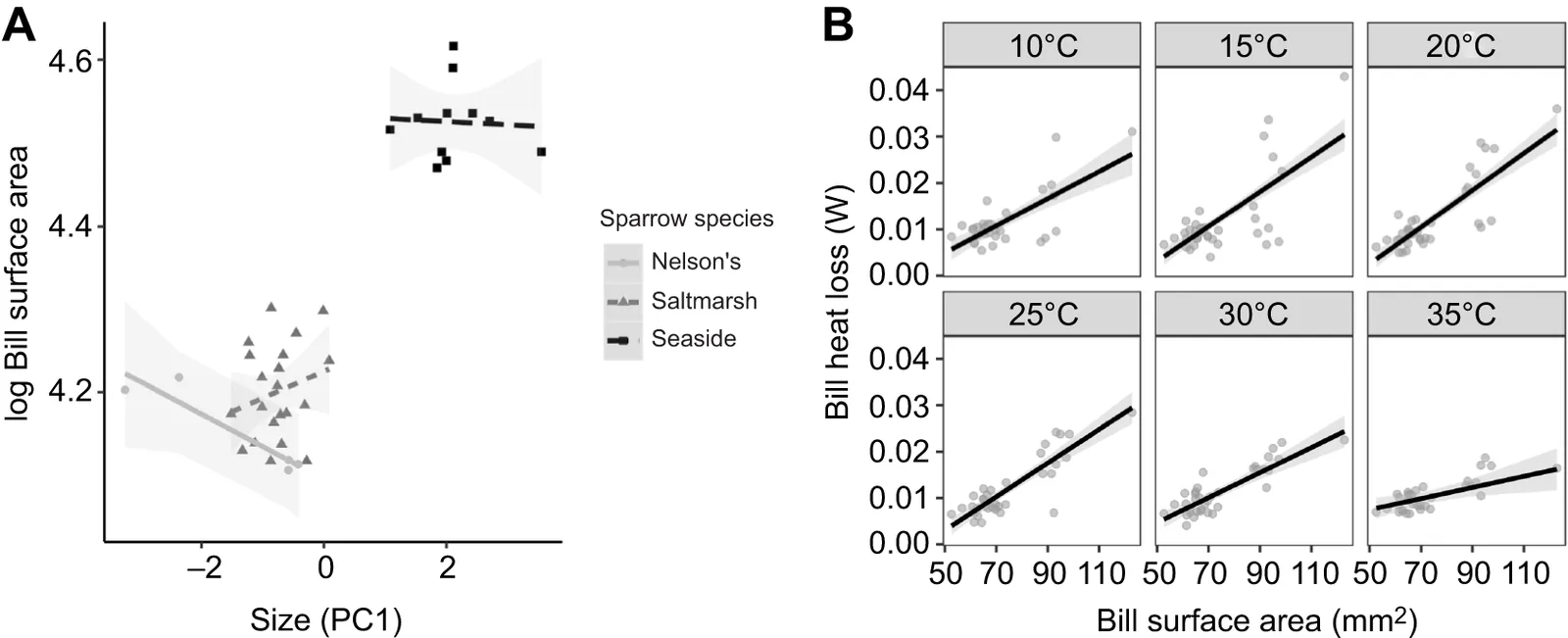
**Species differ in bill allometry, yet among species, larger bills dissipate more heat in a temperature-dependent manner.** (A) Relationship between body size (principal component 1, PC1) and log-transformed bill surface area (mm^2^) in Nelson's sparrows (*n*=5 individuals), saltmarsh sparrows (*n*=20) and seaside sparrows (*n*=11). Points represent individual birds (biological replicates). Lines show species-specific linear mixed model predictions, shaded regions indicate 95% CI. (B) Relationship between bill surface area and bill heat loss across experimental temperatures (10–35°C). Points represent repeated thermographic measurements; lines show predictions from linear mixed models and shaded regions indicate 95% confidence intervals. Repeated thermographic measurements within individuals were treated as repeated measures and not as independent samples.

#### Do larger-billed birds lose more heat just because they have a larger radiator?

The best-supported model for predicting total heat lost from the bill included interactions between bill surface area and both the linear and quadratic terms for *T*_a_ (Table 3; Table S8), indicating that the relationship between bill surface area and heat loss varied with ambient temperature. The second-ranked model additionally included an interaction between species and bill surface area but was less supported (ΔAICc=2.43). Larger bills were associated with greater heat loss across the full range of exposure temperatures, with the strongest relationship at 20°C, where a 10 mm^2^ increase in bill surface area was associated with a 0.004 W increase in heat loss (95% CI: 0.003–0.005 W), compared with 0.001 W at 35°C (95%CI: 0.0003–0.002 W; Fig. 4B). This corresponded to an approximately 3.3 times stronger relationship between bill size and heat loss at 20°C than at 35°C (Fig. 4B).

**Table 3.** Model comparison for linear mixed models evaluating whether bill size predicts heat loss from the bill surface in tidal marsh sparrows

Model	*K*	AICc	ΔAICc	AICc Wt
BSA×*T*_a_+BSA×*T*_a_^2^	9	−1788.63	0.00	0.56
BSA×Species+BSA×*T*_a_+BSA×*T*_a_^2^	13	−1786.20	2.43	0.17
Species+BSA×*T*_a_+BSA×*T*_a_^2^	11	−1785.93	2.70	0.14
BSA×*T*_a_+BSA×*T*_a_^2^+BSA×*T*_a_^3^	11	−1784.93	3.70	0.09
BSA×Species+BSA×*T*_a_+BSA×*T*_a_^2^+BSA×*T*_a_^3^	15	−1782.32	6.30	0.02
Species+BSA×*T*_a_+BSA×*T*_a_^2^+BSA×*T*_a_^3^	13	−1782.13	6.49	0.02
BSA×*T*_a_	7	−1768.92	19.71	0.00
Null model	4	−1709.43	79.20	0.00

Models within ΔAICc<20 and the null model are shown; the full model set is provided in Table S8. Bill surface area is abbreviated as BSA.

### Physiological control over bill-mediated heat exchange

#### Thermal efficiency of heat loss from the bill

The best-supported model for thermal efficiency (heat dissipation per unit bill surface area) included interactions between species and both the linear and quadratic terms for *T*_a_ (Table 4). All remaining models had ΔAICc>12.7. Seaside sparrows showed a significant non-linear response of thermal efficiency to *T*_a_ (quadratic trend=−20.99, 95% CI: −30.86 to −11.13); there was no significant evidence of non-linearity for Nelson's (quadratic trend=1.72, 95% CI: −10.84 to 14.27) or saltmarsh sparrows (quadratic trend=6.35, 95% CI: −0.13 to 12.83). Seaside sparrows exhibited significantly greater thermal efficiency than both Nelson's and saltmarsh sparrows from 15 to 25°C (Fig. 5A,B), with the greatest differences at 20°C. At 20°C, thermal efficiency was 214 W m^−2^ for seaside sparrows (95% CI: 191–237 W m^−2^), compared with 152 W m^−2^ for Nelson's sparrows (95% CI: 119–186 W m^−2^) and 127 W m^−2^ for saltmarsh sparrows (95% CI: 110–143 W m^−2^). At 30°C, seaside sparrows continued to exhibit significantly greater thermal efficiency than saltmarsh sparrows (53.2 W m^−2^ greater, 95% CI: 19.3–87.0 W m^−2^), but did not significantly differ from Nelson's sparrows (27.7 W m^−2^ greater, 95% CI: −20.9–76.3 W m^−2^). No significant differences among species were observed at 10 or 35°C. Thermal efficiency did not significantly differ between Nelson's sparrows and saltmarsh sparrows across the experimental temperature range. At 20°C, where differences among species were greatest, seaside sparrows were more thermally efficient and dissipated 40.4% and 68.9% more heat per unit bill surface area than Nelson's and saltmarsh sparrows, respectively.

**Fig. 5.**
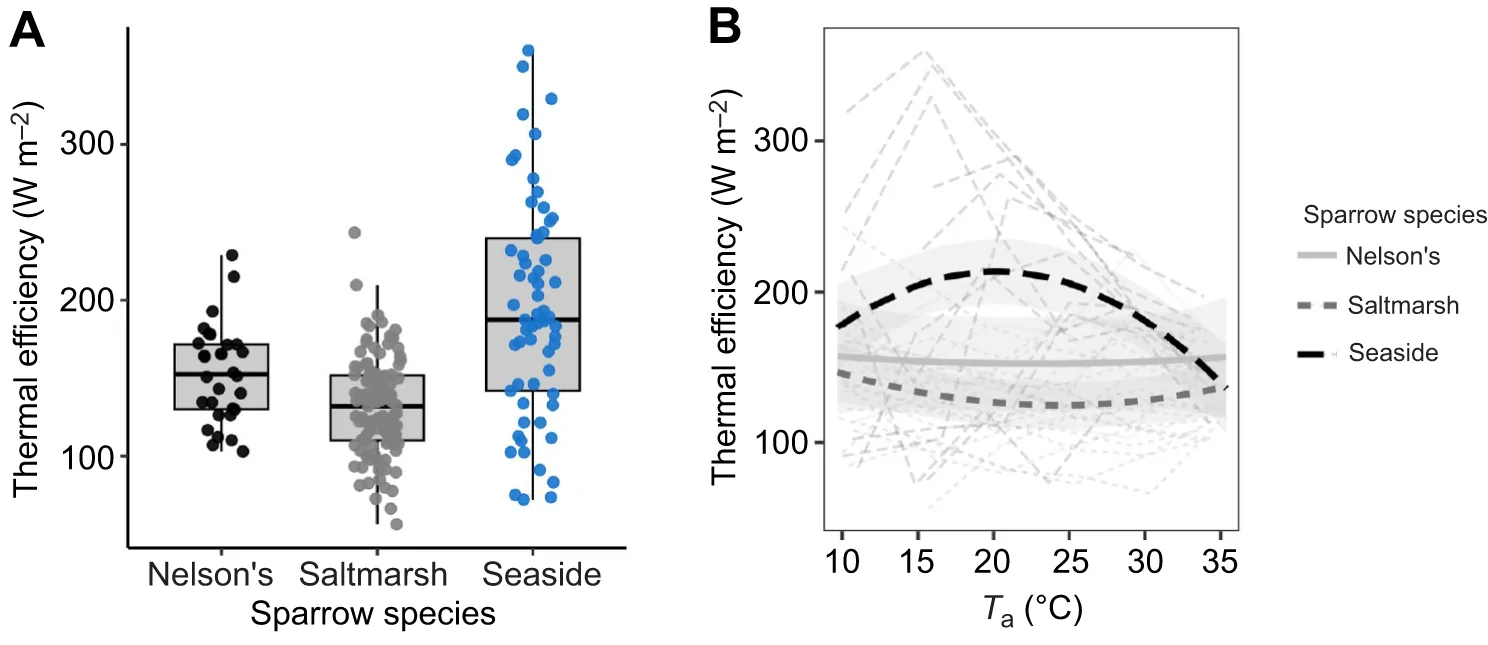
**Seaside sparrows exhibit greater bill thermal efficiency than the other tidal marsh sparrow species.** (A) Thermal efficiency (heat loss per unit bill surface area) in Nelson's sparrows (*n*=5 individuals), saltmarsh sparrows (*n*=20) and seaside sparrows (*n*=11). Boxplots show median, interquartile range and range. Points represent individual thermographic measurements. Species differences were evaluated using linear mixed models (LMMs). (B) Thermal efficiency as a function of ambient temperature for each species. Thin, semi-transparent lines represent observed repeated measurements from individual birds across increasing ambient temperature; thick lines show species-level predictions from linear mixed models (LMMs). Shaded regions indicate 95% CI. Repeated thermographic measurements within individuals were treated as repeated measures and not as independent samples.

**Table 4.** Model comparison for linear mixed models describing bill thermal efficiency (heat loss per unit bill surface area) across tidal marsh sparrow species

Model	*K*	AICc	ΔAICc	AICc wt
Species×*T*_a_+Species×*T*_a_^2^	12	2143.04	0.00	1.00
Species+*T*_a_	7	2155.76	12.72	0.00
Species×*T*_a_	9	2157.41	14.36	0.00
Species+*T*_a_+*T*_a_^2^	8	2157.65	14.61	0.00
Species	6	2157.84	14.80	0.00
*T*_a_	5	2170.48	27.44	0.00
*T*_a_+*T*_a_^2^	6	2172.25	29.21	0.00
Null model	4	2172.63	29.58	0.00

#### Contribution of physiological differences between species in bill-mediated heat loss

For seaside sparrows, the segmented model was better supported than the linear model (ΔAICc=2.5), with a breakpoint in the relationship between bill surface temperature and ambient temperature at approximately 21.2°C (95% CI: 15.8–26.6°C; Fig. 6A). Prior to this breakpoint, the bill surface temperature increased with ambient temperature at a rate of 1.1°C per 1°C (95% CI: 1.0 to 1.2°C) increase in ambient temperature. Above the breakpoint, the slope decreased to 0.8°C per 1°C (95% CI: 0.7°C to 1.0°C) increase in ambient temperature (Fig. 6A), indicating a shallower relationship at higher temperatures. In contrast, there was little evidence for similar physiological control in saltmarsh or Nelson's sparrows. For saltmarsh sparrows, the segmented model was better supported than the linear model (ΔAICc=8.5), but the relationship became slightly steeper above the estimated breakpoint of 17.2°C (95% CI: 13.6–20.7°C; Fig. 6B), increasing from 0.9°C per 1°C (95% CI: 0.86–0.97°C) below the breakpoint to 1.0°C per 1°C (95% CI: 1.00–1.04°C) above it. For Nelson's sparrows, the linear model was better supported than the segmented model (ΔAICc=6.0), providing no evidence for a breakpoint in the relationship between bill surface temperature and ambient temperature (Fig. 6C). Exploratory analyses using ordinary segmented regressions did not support more than one breakpoint for any species in the relationship between bill surface temperature and *T*_a_.

**Fig. 6.**
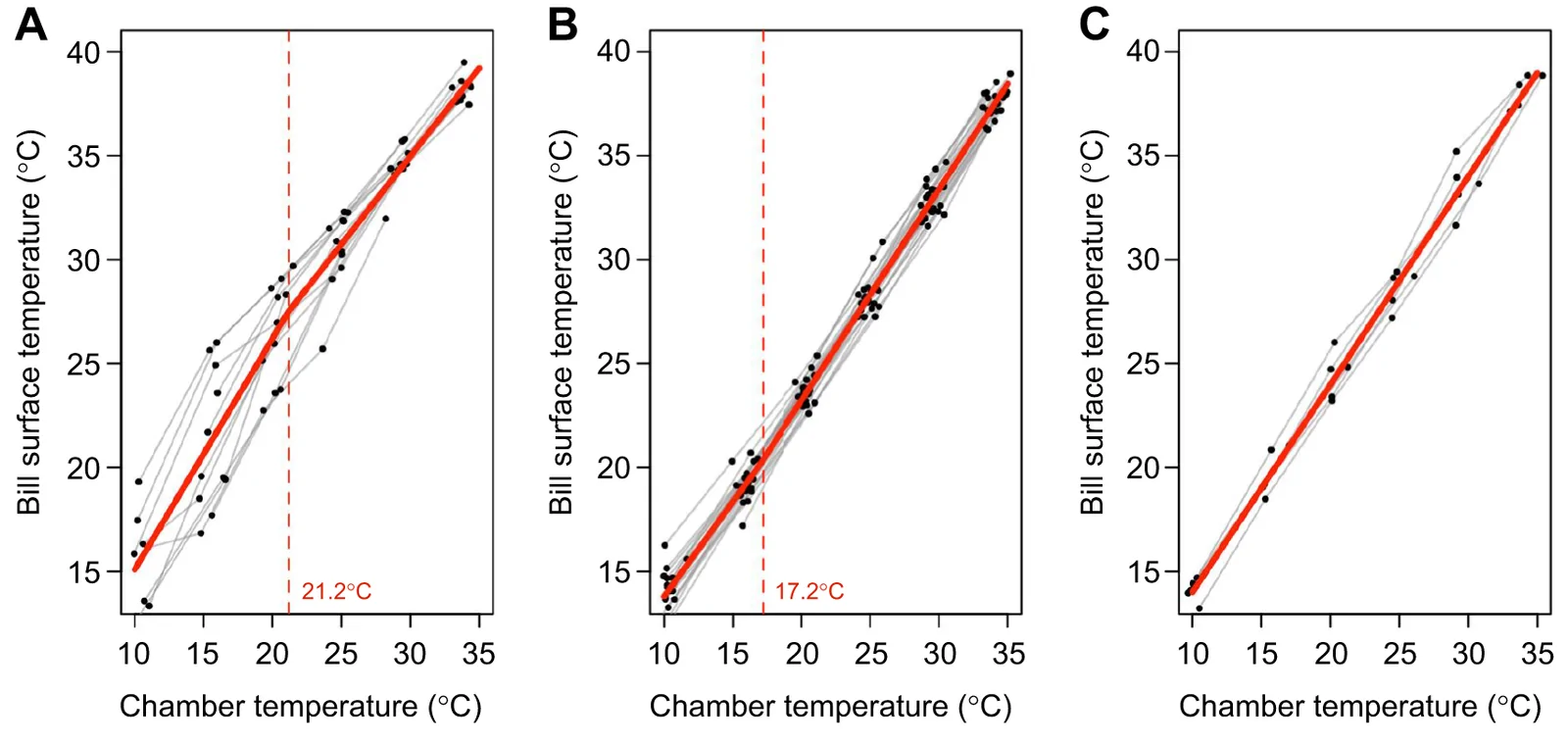
**Species differ in thermal breakpoints of bill surface temperature in response to increasing chamber temperature.** Breakpoint regression plots showing the relationship between chamber temperature and bill surface temperature for (A) seaside sparrows (*n*=11 individuals), (B) saltmarsh sparrows (*n*=20) and (C) Nelson's sparrows (*n*=5). Black points represent individual thermographic measurements; gray lines connect repeated measurements from the same bird. Red solid lines show fitted relationships from LMMs dashed vertical lines indicate estimated breakpoint temperatures at which the slope of bill surface temperature versus chamber temperature changes. Estimated breakpoints were 21.2°C for seaside sparrows and 17.2°C for saltmarsh sparrows. For Nelson's sparrows, the linear model was better supported so no breakpoint is shown. Breakpoints were estimated using segmented LMMs. Repeated measurements within individuals were treated as repeated measures and not as independent samples.

Leg surface temperature, as expected, showed a more complex non-linear relationship with ambient temperature compared with the bill, although evidence for discrete breakpoints was weaker (Fig. S4). For seaside sparrows, the linear and segmented models received similar support (ΔAICc=0.2), with the segmented model estimating a breakpoint at 29.7°C (95% CI: 24.7–34.8°C). Below the estimated breakpoint, leg surface temperature increased at 1.2°C per 1°C increase in *T*_a_ (95% CI: 1.1–1.2°C), compared with 0.9°C per 1°C (95% CI: 0.6–1.2°C) above the breakpoint. Exploratory analyses using ordinary segmented regressions estimated two potential breakpoints at 14.7°C (95% CI: 7.4–22.0°C) and 28.2°C (95% CI: 24.0–32.4°C), with linear, one-breakpoint and two-breakpoint exploratory models receiving similar support (ΔAICc≤1.9). Below the first estimated breakpoint, leg surface temperature increased at 0.9°C per 1°C increase in *T*_a_ (95% CI: 0.3–1.5°C). Between breakpoints, the slope increased to 1.2°C per 1°C (95% CI: 1.1–1.4°C), before decreasing again to 0.9°C per 1°C (95% CI: 0.6–1.1°C) above 28.2°C. For saltmarsh sparrows, the linear model was better supported than the segmented model (ΔAICc=2.4), providing little evidence for a breakpoint in the relationship between leg surface temperature and ambient temperature. For Nelson's sparrows, the linear and segmented models received similar support (ΔAICc=0.4), with the segmented model estimating a breakpoint at 28.7°C (95% CI: 24.5–32.9°C). Below the estimated breakpoint, leg surface temperature increased at 1.2°C per 1°C increase in *T*_a_ (95% CI: 1.1–1.2°C), compared with 0.9°C per 1°C (95% CI: 0.6–1.1°C) above the breakpoint. Exploratory analyses using ordinary segmented regressions provided little evidence for additional thresholds in saltmarsh or Nelson's sparrows: the two estimated breakpoints for saltmarsh sparrows were separated by less than 1°C, while the two-breakpoint model for Nelson's sparrows received substantially less support than the linear model (ΔAICc=7.5).

## DISCUSSION

The bill size of birds in saline environments aligns with predictions of Allen's rule, but appears to be driven by a different thermal extreme than is typically observed in passerines. Passerine bill size is generally better predicted by extreme low rather than high temperatures, but in saline environments the reverse is true. The Greenberg–Tattersall corollary describes this morphological pattern, and is consistent with the idea that high thermal challenges in saline ecosystems impose stronger selection on bird bills than they do in less water-limited landscapes, as a result of the additional osmoregulatory challenges and the advantage of conductive or convective bill thermoregulation over evapotranspiration. We hypothesized that tidal marsh sparrows use their bills as thermal radiators and that interspecific and intraspecific variation in heat dissipation would be linked to bill size.

Our findings confirm for the first time that the bills of three species of tidal marsh sparrow – seaside, saltmarsh and Nelson's sparrows – function as effective thermal radiators under increasing temperatures, with significant differences in the absolute and relative contributions of the bill to total heat loss both within and among taxa (Fig. 3). Seaside sparrows (which are the most specialized of the three taxa to tidal marsh environments) exhibited the highest rates of bill-mediated heat dissipation over much of the experimental temperature range, with the hottest bill surface temperatures, the greatest proportion of total heat lost via the bill, and the highest thermal efficiency, losing more heat per unit bill area than the other species (Fig. 5), particularly at intermediate temperatures. Larger bill size was a key factor in facilitating greater heat loss both within and among species, although the strength of this relationship varied with ambient temperature (Fig. 4). Interestingly, contrary to our initial predictions, seaside sparrows also exhibited evidence of more advanced physiological control over bill-mediated heat loss (Fig. 6). Their bill surface temperature increased non-linearly with ambient temperature, reaching a thermal breakpoint at 21.2°C, beyond which the increase in bill surface temperature slowed, suggesting vasomotor regulation of blood flow – only the second such finding for a passerine (Tattersall et al., 2018). In contrast, saltmarsh and Nelson's sparrows showed more linear heat dissipation patterns and generally lower rates of heat loss indicating more passive thermoregulation typical of the patterns reported more widely among passerines (McQueen et al., 2023). Their bill surface temperatures remained consistently elevated above ambient, suggesting more passive heat dissipation rather than dynamic physiological control.

These interspecific differences align with Allen's rule, which predicts larger appendages in warmer environments to enhance heat dissipation, and with the Greenberg–Tattersall corollary, which suggests that increased bill size is particularly advantageous in freshwater-limited environments (Greenberg et al., 2012a,b; Olsen et al., 2019). At 25°C, seaside sparrows dissipated 141% and 104.4% more heat through their bills than saltmarsh and Nelson's sparrows, respectively, equivalent to differences in dry heat loss of 11.6 and 10.1 mW. Using the latent heat of vaporization of water (2418 J g^−1^; Blaxter, 1986; Greenberg et al., 2012b), these differences correspond to potential water savings of 17.3 mg H_2_O h^−1^ relative to saltmarsh sparrows and 15.1 mg H_2_O h^−1^ relative to Nelson's sparrows, equivalent to approximately 13.7% and 12.0%, respectively, of the expected evaporative water loss of a similarly sized sparrow at 25°C (Bartholomew and Cade, 1963). These estimated water savings exceed those reported by Greenberg et al. (2012b), who found that the greater bill-mediated heat dissipation of a saltmarsh-adapted song sparrow relative to an inland subspecies corresponded to an estimated water saving of 9.7 mg H_2_O h^−1^, or approximately 7.7% of expected evaporative water loss. These findings suggest that species-level differences in bill morphology translate into meaningful variation in thermoregulatory capacity among tidal marsh sparrows. Seaside sparrows, with the largest bills and highest thermal efficiency, dissipated the most heat and had the greatest potential water savings, suggesting an enhanced ability to use the bill for heat loss. Although saltmarsh and Nelson's sparrows also exhibited substantial bill-mediated heat dissipation, their generally lower rates of heat loss suggest a more limited capacity for bill-mediated thermoregulation than seaside sparrows, which may be compensated for by alternative physiological or behavioral mechanisms.

The rate at which surface temperature increases in thermoregulatory windows, such as legs and bills, can provide evidence of advanced physiological control (Tattersall et al., 2018). Comparative studies of live and dead birds show that, in the absence of physiological regulation, bill and leg temperatures rise passively with ambient temperature. However, in live birds, active regulation via vasoconstriction and vasodilation allows appendage surface temperatures to be modulated relative to environmental conditions, regulating heat exchange across a range of ambient temperatures (Tattersall et al., 2018). While both legs and bills function as thermal windows, McQueen et al. (2023) found that legs exhibit stronger physiological control, dynamically adjusting heat exchange, whereas bill heat dissipation is often more passive. Our results generally support this pattern: leg surface temperature increased with ambient temperature across all species, with evidence of a complex non-linear relationship in seaside sparrows (Fig. S4), consistent with more advanced physiological control at both low and high temperatures.

While McQueen et al. (2023) did not detect a similar sigmoidal pattern in bills, suggesting that bill heat loss is often more passive, our findings indicate that bill-mediated heat dissipation in tidal marsh sparrows, particularly in seaside sparrows, may be more dynamically regulated than previously recognized. Seaside sparrows exhibited evidence of physiological control over bill-mediated heat loss. Though their bill surface temperatures did not dip below plumage surface temperature, they approached it at lower exposure temperatures, suggesting some reduction in heat transfer (Fig. S1). Their bill surface temperature then showed a significant non-linear increase with ambient temperature, as compared with the more linear increases of the other two species, with bill surface temperature remaining elevated above body surface temperature throughout the exposure period. This non-linear relationship between bill surface temperature and ambient temperature, combined with their significantly higher thermal efficiency across intermediate temperatures, suggests that seaside sparrows may exert more precise physiological control over heat loss from the bill compared with saltmarsh and Nelson's sparrows and, indeed, more than any previous passerine examined that we are aware of (although see Tattersall et al., 2018). Breakpoint regression analysis further supported this non-linear pattern and revealed a thermal breakpoint at 21.2°C, beyond which the slope of bill surface temperature increase flattened (Fig. 6). These findings suggest active regulation, likely through mechanisms such as vasodilation, enabling rapid heat dissipation as ambient temperatures rise. However, the capacity for bill-mediated heat loss is inherently limited by the thermal gradient between the bill and the surrounding air. Once ambient temperature approaches or surpasses bill surface temperature, this gradient diminishes, reducing the potential for dry heat transfer even if blood flow to the bill remains elevated. This explains why the greatest differences in heat dissipation and thermal efficiency occurred at intermediate rather than the highest experimental temperatures.

In contrast, saltmarsh and Nelson's sparrows exhibited more linear relationships between bill surface temperature and ambient temperature, suggesting less dynamic regulation of heat dissipation. Although a breakpoint model was supported for saltmarsh sparrows, the difference in slopes before and after the breakpoint was small, and the slope remained positive, providing little evidence of the same pattern observed in seaside sparrows. Interestingly, both saltmarsh and Nelson's sparrows maintained bill surface temperature at or below their plumage surface temperature at the lowest exposure temperature (10°C; Fig. S1), consistent with heat conservation strategies. Only at ambient temperatures exceeding 20–25°C did their bill surface temperature rise above plumage temperature, increasing approximately linearly with ambient temperature. Bill surface temperature in both species remained elevated above ambient temperature throughout the duration of the trial, increasing by approximately 1°C with every 1°C increase in ambient temperature.

This raises an important question: if seaside sparrows' bills are adapted to maximize heat loss in high temperatures, how do they avoid the disadvantage of excessive heat loss in colder conditions predicted by Allen's rule (Tattersall et al., 2016)? We hypothesize that their non-linear relationship between bill surface temperature and ambient temperature may hold the answer. Tattersall et al. (2018) showed that Darwin's finches (*Geospiza* spp.) with relatively large bills did not face greater thermoregulatory challenges at either thermal extreme; instead, their bills lost less heat per unit area at low temperatures and gained less heat per unit area at high temperatures, suggesting an adaptive mechanism to balance dissipation and conservation. We observed a similar pattern in seaside sparrows: their non-linear relationships with temperature and thermal efficiency (heat loss per unit bill area) support the idea that larger-billed birds optimize dissipation in warm conditions while minimizing loss in cooler environments. At the extreme ends of our exposure range, seaside sparrow thermal efficiency did not differ significantly from that of the other taxa (confidence intervals overlapped), but at mid-range temperatures, they exhibited significantly higher thermal efficiency. Notably, 4 of the 11 seaside sparrows in our study lost 4.3–5.6% of their total body heat via the bill, surpassing even *Geospiza magnirostris* in the Tattersall et al. (2018) study, which lost 4.3%. The mean and median bill-mediated heat loss among seaside sparrows were 3.35% and 3.37%, respectively. These rates of loss are still disproportionate, as the bill represents only approximately 2% of the total surface area of each of the three *Ammospiza* species, compared with 4% in *G. magnirostris* (Tattersall et al., 2018). These estimates are also likely conservative, as thermal models tend to overestimate heat retention in plumage-insulated regions while underestimating the contribution of exposed, non-insulated structures such as the bill (Tattersall et al., 2018).

The differences among species may be partly explained by the distinct thermal environments they occupy during the breeding season and their geographic distributions. Nelson's sparrows occur furthest north, breeding in the Acadian coastal zone, where both ambient and weekly thermal environments during our trials were the coolest. Across all individual Nelson's sparrows measured, daily mean temperature experienced by individuals on the day of their thermal trial averaged 16.1°C (range 13–21.1°C), and mean temperatures from the week preceding the trials averaged 18.2°C (range 16.4–19.5°C). Saltmarsh sparrows exhibited intermediate temperature conditions of the three species tested, consistent with their broader geographic distribution (Maine to Virginia, USA) and occupancy of both northern and more southerly marshes where they co-occur with seaside sparrows. They experienced mean daily temperatures of 22.7°C (range 15–27.9°C) and mean weekly temperatures of 23.7°C (range 18.8–28°C). Seaside sparrows, despite being sampled at the northernmost edge of their breeding range, experienced the warmest conditions of the three species during thermography trials, with mean daily temperatures of 23.4°C (range 18–28°C) and mean weekly temperatures of 25.7°C (range 22–28.9°C). It is important to note that our sampling scheme covered roughly two-thirds of the breeding range for saltmarsh sparrows and the southern half of the breeding range for Acadian Nelson's sparrows, while only representing the northern limit of the seaside sparrow breeding range. The mean daily values we present here for seaside sparrows likely underestimate the thermal challenges faced by resident seaside sparrows in the southern portions of their range, where environmental temperatures are considerably higher.

### Limitations and future directions

Despite strong evidence of active bill-mediated thermoregulation in seaside sparrows, several limitations of our study should be acknowledged, along with key areas for future research. Our experimental design exposed sparrows to a temperature range of 10–35°C, capturing typical summer ambient conditions but not necessarily the full extent of their thermoregulatory capacity. Expanding this temperature range to include both lower and higher temperatures would provide a more comprehensive assessment of heat dissipation strategies and potential heat conservation mechanisms. Additionally, direct physiological measurements of water loss are needed to determine how increased heat dissipation from the bill interacts with evaporative cooling, a key prediction of the Greenberg–Tattersall corollary. Future studies integrating water loss measurements with thermal imaging would help clarify the functional trade-offs between bill-mediated heat dissipation and evaporative cooling.

Although not a primary focus of this study, we observed several thermoregulatory behaviors that varied across species during the trials. Panting was relatively rare across all taxa, even at 35°C, a temperature that approaches typical avian body temperature (*T*_b_) and at which panting is generally expected to be initiated (Angilletta, 2009). This absence was especially notable in seaside sparrows, which rarely panted, consistent with their generally higher rates of bill-mediated heat loss and greater thermal efficiency. In contrast, panting was occasionally observed in saltmarsh and Nelson's sparrows at the highest temperature exposure. Ruffling of feathers occurred in all species and increased with temperature, suggesting a shared behavioral response to heat stress. Interestingly, seaside sparrows frequently exhibited yawning at higher temperatures, a behavior that has been linked to thermoregulation in other avian species, including budgerigars (*Melopsittacus undulatus*; Gallup et al., 2009). Further investigation into the thermoregulatory function of yawning, particularly in species that appear to rely less on panting, may offer new insights into alternative mechanisms of heat dissipation in birds.

Additionally, while our study focused on males, future research should investigate female thermoregulatory strategies, particularly in relation to seasonal shifts in thermal selection pressures. Given the sexual dimorphism in bill morphology observed in seaside sparrows (Greenberg and Olsen, 2010), males and females may experience different thermoregulatory challenges. Future work comparing the thermoregulatory responses of both sexes across breeding and non-breeding seasons would provide valuable insights into sex-specific selection pressures shaping bill morphology.

Finally, while our findings demonstrate that larger bills serve as effective thermal radiators, the precise physiological mechanisms underlying bill-mediated heat dissipation remain unclear. Seaside sparrows exhibited non-linear responses in bill surface temperature, heat loss and thermal efficiency suggesting active vasomotor regulation, yet the specific vascular mechanisms remain unknown. Our findings, specifically in seaside sparrows, lead us to suggest there may be physiological controls that lessen the tradeoff between winter and summer thermoregulation in at least this one species, such that the ability to lose heat to the environment during warm periods is not necessarily accompanied by the negative effects of high heat loss during cold periods as well. Direct tests of this over a wider temperature range than we examined here, and with birds that are cold acclimated during the non-breeding months, would be required to test this explicitly. In particular, comparative studies using infrared thermography alongside vascular imaging techniques could help elucidate how blood flow regulation contributes to bill-mediated heat exchange under all these conditions.

### Conclusions

Our study demonstrates that, among tidal marsh sparrows, bill morphology plays a critical role in thermoregulation with important implications for water conservation. Across the three species, larger bills facilitated greater heat dissipation (the strength of this relationship varying with ambient temperature), providing the potential for greater water savings and reinforcing the adaptive significance of bill size in thermally and hydrically stressed environments. However, the non-linear thermal response observed in seaside sparrows provides evidence of physiological control of bill-mediated heat dissipation suggesting that this is not a strictly passive process and potentially challenging the idea that appendages such as the bill function primarily as static thermal radiators. Instead, our findings add to a growing body of evidence suggesting that at least some bird species exert active physiological regulation over bill temperature, much like the legs, which are well-documented sites of vascular control (McQueen et al., 2023; Tattersall et al., 2018).

Our study also provides insight into the evolutionary pressures shaping avian appendages beyond classic ecogeographic rules. While Allen's rule and the Greenberg–Tattersall corollary predict larger appendages in warmer or freshwater-limited environments to facilitate dry heat loss, our findings suggest that selection may also favor physiological adaptations that optimize bill function across different thermal contexts. Although our study focused on tidal marsh sparrows, similar selective pressures likely act on other avian taxa without salt glands that are exposed to extreme heat or freshwater scarcity. Understanding whether physiological control of bill temperature is widespread or limited to certain species with particularly high thermal loads and water stress is an important avenue for future research. Given the increasing frequency of extreme heat events under climate change, further studies examining the plasticity and adaptability of bill-mediated thermoregulation will provide critical insights into how birds may cope with rising global temperatures. By expanding our understanding of how avian appendages contribute to thermoregulation, this study builds upon recent research illustrating that the evolution of avian bills is dictated not solely by feeding ecology but also by thermoregulatory demands (Tattersall et al., 2016). Our findings suggest that bill morphology should be considered a key component of avian thermal adaptation strategies, with implications for predicting how birds will respond to environmental change in the future.

## Supplementary Material

10.1242/jexbio.252510_sup1 Supplementary information
