# Supplementary material for: Bill morphology and species identity predict heat loss at high air temperatures among tidal marsh sparrows in saline environments
Source: J Exp Biol. 2026 Oct 14;229(19):jeb252510. doi: 10.1242/jeb.252510 (PMC13723393; doi:10.1242/jeb.252510)
Supplement: Supplementary file 1 [file jexbio-229-252510-s1.pdf]

Supplementary Materials and Methods

Appendix 1

Thermal Chamber Specifics

The chamber features an AC-162 thermoelectric cooling assembly (hereafter TCA; TE Technology, Traverse City, Michigan, USA) mounted into the lid of a 50-quart, wheeled, Coleman portable cooler (Coleman Company, Inc., Kingfisher, Oklahoma, USA; dimensions: 57.4 cm x 44.7 cm x 44.5 cm, see Fig. 2), connected to a 24-volt DC fixed voltage power supply (TE Technology, Traverse City, Michigan, USA) and a TC-720 bipolar thermoelectric temperature controller (TE Technology, Traverse City, Michigan, USA), powered by a Jackery Explorer 1000 portable power station (Jackery, Fremont, California, USA). An MP-3193 moisture-resistant thermistor (TE Technology, Traverse City, Michigan, USA), connected to the TCA and controller, hangs in the center of the chamber interior, and communicates internal conditions to the temperature controller. The interior of the chamber contains a smaller bird-housing chamber: a 5.2L plastic container (dimensions: 22.6 cm x 18.7 cm x 18.7 cm) with a wire mesh lid and evenly spaced drilled holes along the lower half of each wall for air flow, a metal grate, and a front-mounted CorDEX IW3000 Smart 7.6 cm Infrared (IR) viewing window (CorDEX, Scarborough, England, United Kingdom). The viewing window abuts a 7.6 cm hole in the front cooler wall where a Forward Looking InfraRed (FLIR) E-95 IR (464 x 348 resolution) camera (Teledyne FLIR, Wilsonville, Oregon, United States) with a 42° lens is placed to record the bird inside the chamber, mounted on a JOBY Gorillapod 3K tripod stand (JOBY, Petaluma, California, United States). A cylindrical piece of light-blocking fabric around the hole on the cooler's exterior surface allows for camera placement and bird observation while preventing external light from entering the chamber. On either side of the bird-housing chamber within the cooler we placed a professionally calibrated, ground-level, Tempo Disc Bluetooth-enabled thermometer, hygrometer, and dew point logger (BlueMaestro, Montgomery, Texas, United States) to record the chamber climatic conditions.

Testing for Confounding Influences on Surface Temperature and Heat Loss

To account for potential confounding factors (such as activity rate, local climatic acclimation, and variation in relative humidity between trials) that could influence ROI surface temperatures and heat loss, we conducted a series of analyses.

Journal of Experimental Biology • Supplementary information

Activity – First, we examined the relationship between activity and ambient temperature (T_a). Activity, such as hopping, could increase metabolic heat production, thereby influencing surface temperatures. Although the chamber was designed with confined dimensions and a darkened interior to minimize bird movement during the trial, some activity still occurred. To account for this, we quantified an activity rate for each bird by counting the total number of hops (movements involving both feet leaving the ground) during the exposure period, providing a measure of hops per minute (hereafter activity rate) at each temperature. We fitted a linear mixed-effects model (LMM) using the *lme4* package (Bates et al., 2015), treating individual identity as a random effect and including ambient temperature (T_a), the squared term of T_a (T_a^2), species, and their interactions as fixed effects. Activity rate was log-transformed prior to analysis to account for its strongly right-skewed distribution. We defined T_a as the mean value of T_a measured in the thermal chamber over the final exposure minute for each exposure temperature within the trial. Model fit was assessed using residual-versus-fitted and normal Q-Q plots. The model showed considerable variability in activity across individuals (random intercept variance = 1.93, SD = 1.39). Because our model indicated a high level of individual variation in activity rate, and activity could influence metabolic heat production and surface temperature, we proceeded with our subsequent analyses, including activity rate as a fixed effect in every model used in model comparison.

Local Temperature Acclimation

Additionally, we tested the potential impacts of local temperature acclimation on surface temperature for birds tested at different geographic locations and times during the breeding season. This was necessary to ensure that any observed differences in bill surface temperature were due to intrinsic species characteristics or thermal conditions during the trial, rather than short-term acclimatization to the local environment prior to capture. To determine if any of our acclimation

Journal of Experimental Biology • Supplementary information

variables impacted ROI surface temperature we generated a series of linear mixed models for comparison. All models included individual identity as a random variable, and internal chamber T_a and species as fixed variables to control for the proximal surface temperature changes associated with the exposure trial and for any species-level bill temperature differences, respectively. Our local temperature acclimation variables included trial date, the local temperature recorded in the hour prior to the bird's capture, as well as a weekly average temperature from the bird's location. We tested all combinations of these acclimation variables and included interactions with species for each term in each model. A null model, excluding acclimation variables, was also generated for comparison, and model performance was evaluated using AIC*c* scores with the *aictab* function from the *AICcmodavg* package (Mazerolle, 2020). Among the eight candidate models tested, the null model, which included individual identity as a random effect and chamber temperature and species as fixed effects, had the lowest AICc value (646.46) and the highest Akaike weight (0.75, see Table S3). The second-best model had a ΔAICc of 4.19, greater than 2, indicating that none of the local acclimation variables explained ROI surface temperatures during the trial better than the null without them. As a result, we did not consider local temperature acclimation variables to impact our surface temperatures of interest, and these variables were excluded from subsequent analyses.

Variation in Relative Humidity among Trials

We also assessed whether variation in environmental conditions within the chamber, specifically humidity, could influence surface temperature measurements across trials. Although the chamber was designed to maintain consistent temperature and humidity exposures, we sought to confirm this consistency by modeling the relative humidity (calculated as the average across each minute of the exposure period) as a function of T_a, species, and their interaction. We compared AICc scores to identify the best-fitting model. As expected, T_a was the strongest predictor of chamber humidity (AICc = 200.25,

Journal of Experimental Biology • Supplementary information

see Table S4), with relative humidity showing a consistent negative correlation with T_a across all trials. Due to this consistent relationship, and the lack of influence from other variables, measures of relative humidity were excluded from subsequent analyses.

Table S1. Thermography sampling locations. Geographic locations and sample sizes of tidal marsh sparrows included in a thermographic study along the northern Atlantic Coast of the United States.

State	Marsh Name	Latitude, Longitude	N Birds (by Species)
Maine	Rossport	44.93°, -67.04°	Nelson's sparrow: 2
	Lubec	44.82°, -66.99°	Nelson's sparrow: 2
New Hampshire	Chapman's Landing	43.04°, -70.93°	Saltmarsh sparrow: 8<br>Nelson's sparrow: 1
Connecticut	Hammonasset Beach State Park	41.26°, -72.55°	saltmarsh sparrow: 3
New Jersey	Edwin B. Forsythe National Wildlife Refuge - Oyster Creek	39.50°, -74.43°	seaside sparrow: 4<br>saltmarsh sparrow: 3
	Edwin B. Forsythe National Wildlife Refuge - ATT	39.70°, -74.26°	saltmarsh sparrow: 6<br>seaside sparrow: 7

Table S2. Morphological comparisons among tidal marsh sparrows. Comparison of Nelson's sparrows (NESP), saltmarsh sparrows (SALS), and seaside sparrows (SESP). Presented values are means with standard deviations in parentheses. P-values represent pairwise comparisons following ANOVA or Welch's ANOVA (in cases where Levene's test indicated a violation of homogeneity of variances, marked with "*"), with post-hoc Tukey's HSD or Games-Howell tests for pairwise comparisons. Significant differences (P < 0.05) following multiple comparison corrections are highlighted in bold.

Measurement	Nelson's Sparrow (5)	Saltmarsh Sparrow (20)	Seaside Sparrow (11)	P-value (NESP vs SALS)	P-value (NESP vs SESP)	P-value (SALS vs SESP)
Wing chord (mm)	58.2 (2.2)	57.5 (1.0)	61.4 (1.3)	**<0.01**	**<0.01**	**<0.01**
Mass (g)	17.3 (1.7)	19.8 (1.0)	22.8 (0.9)	**<0.01**	**<0.01**	**<0.01**
Tarsus (mm)	21.1 (1.2)	21.4 (0.3)	23.6 (0.8)	0.8*	**0.01**	**<0.01**
Distal bill area (mm^2)	63.6 (3.5)	66.8 (3.8)	92.4 (4.3)	0.3	**<0.01**	**<0.01**
Tarsus area (mm^2)	194.1 (28.1)	203.9 (12.3)	244.2 (22.2)	0.5	**<0.01**	**<0.01**
Body area (mm^2)	5463.2 (354.1)	5982.1 (208.6)	6589.9 (180.2)	**<0.01**	**<0.01**	**<0.01**
Distal Bill area (% of total)	1.98 (0.2)	1.93 (0.1)	2.3 (0.1)	0.8	**0.01**	**<0.01**
Tarsus area (% of total)	3.4 (0.5)	3.2 (0.3)	3.6 (0.4)	0.7	0.6	0.1
Body area (% of total)	94.7 (0.6)	94.8 (0.3)	94.2 (0.4)	0.7	0.1	**<0.01**
Wing chord (mm)	58.2 (2.2)	57.0 (1.0)	61.4 (1.3)	**<0.01**	**<0.01**	**<0.01**

Journal of Experimental Biology • Supplementary information

Table S3. Linear mixed models describing potential impacts of local temperature acclimation on the measured bill surface temperatures during the trial. Each model included individual as a random variable, and T_a and species as fixed variables to control for the proximal surface temperature changes associated with the exposure trial and for any species-level bill temperature differences, respectively. Fixed variables of interest included trial date, the local temperature recorded in the hour prior to the bird's capture, as well as a weekly average temperature from the bird's location. We tested all combinations of these acclimation variables and included interactions with species for each term in each model. A null model, excluding acclimation variables, was also generated for comparison. The null model contained only individual as a random variable, and T_a and species as fixed variables.

Model	K	AICc	ΔAICc	AICc Wt
Null Model	6	646.46	0.00	0.75
T_a + Species * Local T_a	9	650.65	4.19	0.09
T_a + Species * Date	9	651.04	4.58	0.08
T_a + Species * Weekly Temp	9	651.54	5.08	0.06
T_a + (Species * Local T_a) + (Species * Weekly Temp)	12	655.16	8.69	0.01
T_a + (Species * Date) + (Species * Local T_a)	12	656.00	9.54	0.01
T_a + (Species * Date) + (Species * Weekly Temp)	12	656.59	10.13	0.00
T_a + (Species * Date) + (Species * Local T_a) + (Species * Weekly Temp)	15	661.73	15.26	0.00

Table S4. Linear mixed models describing the humidity of the thermal chamber during the temperature exposure trials. Variables of interest included T_a and Species, as well as their interaction. Each model included trial as a random variable, the null model was an intercept-only model.

Model	K	AICc	ΔAICc	AICc Wt
T_a	4	200.25	0.00	0.72
T_a + Species	6	203.33	3.08	0.16
T_a * Species	8	203.83	3.57	0.12
Null Model	3	486.82	286.56	0.00
Species	5	489.93	289.68	0.00

Table S5. Is the bill a thermal radiator? Does bill surface temperature change with ambient temperature? Linear mixed models describing the surface temperature of the bill as a function of ambient temperature in the chamber (T_a) and species identity for three tidal marsh sparrow species. All models (including the null) have individual identity as a random effect and activity rate (hops/minute) as a fixed effect.

Model	K	AICc	ΔAICc	AICc Wt
Species * T_a + Species * T_a^2	12	634.21	0.00	0.94
Species * T_a + Species * T_a^2 + Species * T_a^3	15	639.96	5.75	0.05
Species + T_a	7	647.82	13.61	0.00
Species * T_a	9	649.11	14.91	0.00
Species + T_a + T_a^2	8	649.48	15.28	0.00
Species + T_a + T_a^2 + T_a^3	9	651.12	16.91	0.00
T_a	5	667.49	33.28	0.00
T_a + T_a^2	6	669.01	34.80	0.00
T_a + T_a^2 + T_a^3	7	670.63	36.43	0.00
Null Model	4	1471.82	837.61	0.00
Species	6	1473.79	839.59	0.00

Table S6: Linear mixed models describing heat loss (watts) from the bill surface as a function of ambient temperature in the chamber (T_a) and species identity for three tidal marsh sparrow species. All models (including the null) have individual identity as a random effect and activity rate (hops/minute) as a fixed effect.

Model	K	AICc	ΔAICc	AICc Wt
Species * T_a + Species * T_a^2	12	-1759.40	0.00	0.95
Species * T_a + Species * T_a^2 + Species * T_a^3	15	-1753.25	6.15	0.04
Species * T_a	9	-1744.03	15.36	0.00
Species + T_a	7	-1743.33	16.07	0.00
Species + T_a + T_a^2	8	-1742.41	16.99	0.00
Species	6	-1741.25	18.15	0.00
Species + T_a + T_a^2 + T_a^3	9	-1740.52	18.88	0.00
T_a	5	-1711.65	47.75	0.00
T_a + T_a^2	6	-1710.93	48.47	0.00
Null Model	4	-1709.43	49.97	0.00
T_a + T_a^2 + T_a^3	7	-1709.06	50.34	0.00

Journal of Experimental Biology • Supplementary information

Table S7. Does total heat loss from the bill (as a percentage of total body heat loss) vary with ambient temperature? Model comparison results for linear mixed models describing bill heat loss as a percentage of total body heat loss across tidal marsh sparrow species as a function of ambient temperature in the chamber (T_a) and species identity for three tidal marsh sparrow species. All models (including the null) have individual identity as a random effect and activity rate (hops/minute) as a fixed effect.

Model	K	AICc	ΔAICc	AICc Wt
Species * T_a + Species * T_a^2	12	301.21	0.00	0.95
Species * T_a + Species * T_a^2 + Species * T_a^3	15	307.13	5.91	0.05
Species + T_a	7	333.74	32.52	0.00
Species + T_a + T_a^2	8	335.67	34.46	0.00
Species * T_a	9	336.05	34.84	0.00
Species + T_a + T_a^2 + T_a^3	9	337.66	36.45	0.00
Species	6	346.25	45.03	0.00
T_a	5	368.25	67.04	0.00
T_a + T_a^2	6	370.16	68.94	0.00
T_a + T_a^2 + T_a^3	7	372.14	70.92	0.00
Null Model	4	381.45	80.24	0.00

Journal of Experimental Biology • Supplementary information

Table S8. Does bill size predict heat loss? Model comparison results for linear mixed models describing heat loss (in watts) by the bill as a function of bill surface area, ambient temperature in the chamber (T_a), and species identity for three tidal marsh sparrow species. All models (including the null) have individual identity as a random effect and activity rate (hops/minute) as a fixed effect.

Model	K	AICc	ΔAICc	AICc Wt
Bill Surface Area * T_a + Bill Surface Area * T_a^2	9	-1788.63	0.00	0.56
Species * Bill Surface Area + Bill Surface Area * T_a + Bill Surface Area * T_a^2	13	-1786.20	2.43	0.17
Species + Bill Surface Area * T_a + Bill Surface Area * T_a^2	11	-1785.93	2.70	0.14
Bill Surface Area * T_a + Bill Surface Area * T_a^2 + Bill Surface Area * T_a^3	11	-1784.93	3.70	0.09
Species * Bill Surface Area + Bill Surface Area * T_a + Bill Surface Area * T_a^2 + Bill Surface Area * T_a^3	15	-1782.32	6.30	0.02
Species + Bill Surface Area * T_a + Bill Surface Area * T_a^2 + Bill Surface Area * T_a^3	13	-1782.13	6.49	0.02
Bill Surface Area * T_a	7	-1768.92	19.71	0.00
Bill Surface Area * T_a + Species	9	-1766.39	22.24	0.00
Species * T_a + Species * T_a^2	12	-1759.40	29.23	0.00
Species * T_a + Bill Surface Area	10	-1756.19	32.43	0.00
Bill Surface Area	5	-1755.86	32.76	0.00
Species * Bill Surface Area + T_a	10	-1755.83	32.79	0.00
Species + Bill Surface Area + T_a	8	-1755.66	32.97	0.00
Species * Bill Surface Area + T_a + T_a^2	11	-1754.89	33.73	0.00
Species * Bill Surface Area	9	-1753.77	34.86	0.00
Species + Bill Surface Area	7	-1753.49	35.13	0.00
Species * T_a + Species * T_a^2 + Species * T_a^3	15	-1753.25	35.38	0.00
Species * Bill Surface Area + T_a + T_a^2 + T_a^3	12	-1752.98	35.64	0.00
Species * T_a	9	-1744.03	44.59	0.00
Species + T_a	7	-1743.33	45.30	0.00
Species + T_a + T_a^2	8	-1742.41	46.21	0.00
Species	6	-1741.25	47.38	0.00
Species + T_a + T_a^2 + T_a^3	9	-1740.52	48.11	0.00
T_a	5	-1711.65	76.97	0.00
T_a + T_a^2	6	-1710.93	77.69	0.00
Null Model	4	-1709.43	79.20	0.00
T_a + T_a^2 + T_a^3	7	-1709.06	79.57	0.00

Journal of Experimental Biology • Supplementary information

Table S9. Is the body acting as a thermal radiator? Linear mixed models describing surface temperature of the body as a function of ambient temperature in the chamber (T_a, and species identity for three tidal marsh sparrow species. All models (including the null) have individual identity as a random effect and activity rate (hops/minute) as a fixed effect.

Model	K	AICc	ΔAICc	AICc Wt
T_a	5	330.64	0.00	0.33
$T_a + T_a^2 + T_a^3$	7	332.04	1.40	0.16
Species + T_a	7	332.11	1.47	0.16
$T_a + T_a^2$	6	332.63	1.99	0.12
Species * T_a	9	333.31	2.67	0.09
Species + $T_a + T_a^2 + T_a^3$	9	333.59	2.95	0.08
Species + $T_a + T_a^2$	8	334.14	3.49	0.06
Species * T_a + Species * T_a^2	12	338.34	7.70	0.01
Species * T_a + Species * T_a^2 + Species * T_a^3	15	339.28	8.64	0.00
Null Model	4	1455.49	1124.84	0.00
Species	6	1459.69	1129.05	0.00

Table S10. Is the leg acting as a thermal radiator? Linear mixed models describing the surface temperature of the as a function of ambient temperature in the chamber (T_a), and species identity for three tidal marsh sparrow species. All models (including the null) have individual identity as a random effect and activity rate (hops/minute) as a fixed effect.

Model	K	AICc	ΔAICc	AICc Wt
$T_a + T_a^2 + T_a^3$	7	594.93	0.00	0.69
Species + $T_a + T_a^2 + T_a^3$	9	597.51	2.57	0.19
Species * T_a + Species * T_a^2 + Species * T_a^3	15	598.39	3.46	0.12
Species * T_a	9	607.24	12.31	0.00
T_a	5	608.14	13.21	0.00
$T_a + T_a^2$	6	608.96	14.03	0.00
Species * T_a + Species * T_a^2	12	610.47	15.53	0.00
Species + T_a	7	610.73	15.79	0.00
Species + $T_a + T_a^2$	8	611.55	16.62	0.00
Null Model	4	1427.03	832.10	0.00
Species	6	1431.17	836.24	0.00

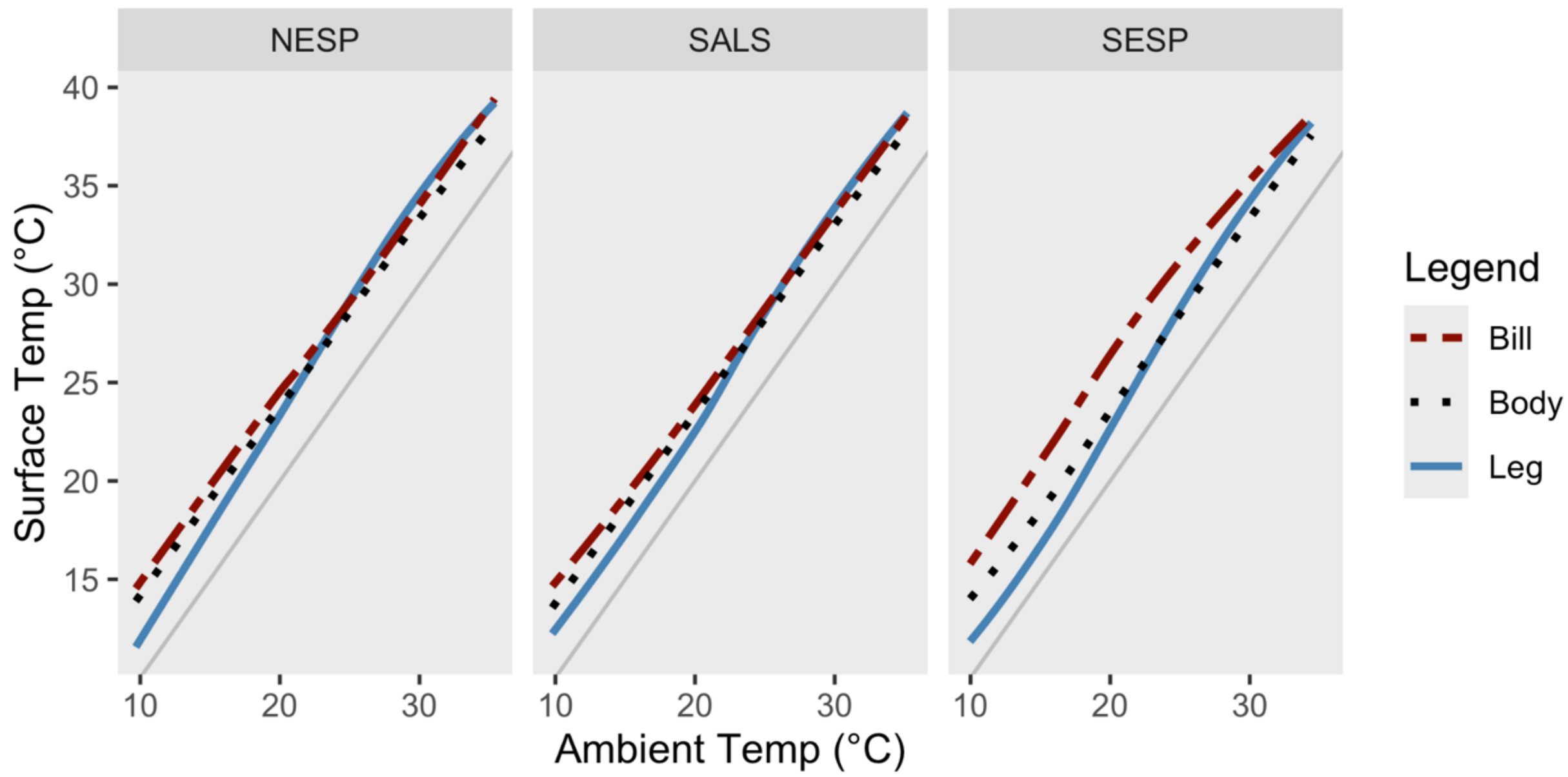

Fig. S1. Relationships between ambient temperature and surface temperature for all regions of interest in tidal marsh sparrows. Predicted surface temperatures (°C) for the bill (red dashed line), body surface (black dotted line), and legs (blue solid line) are shown across the range of ambient temperatures (T_a) used in thermal chamber trials for (A) Nelson's sparrow (NESP), (B) saltmarsh sparrow (SALS), and (C) seaside sparrow (SESP). Lines represent model-predicted means from linear mixed-effects models with individual identity as a random effect. The solid grey 1:1 line ($T_s = T_a$) is shown for reference.

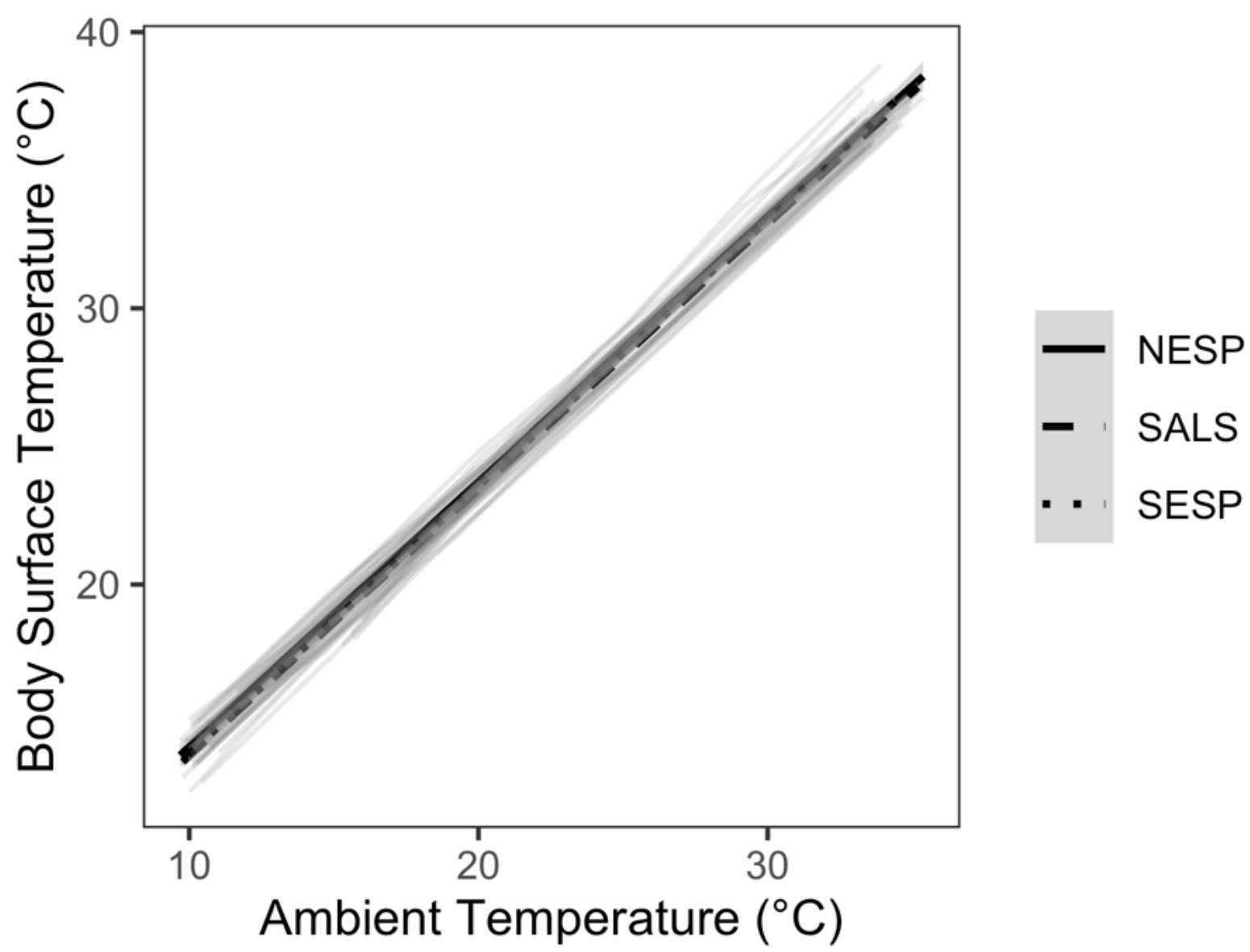

Fig. S2. Body surface temperature of seaside sparrows (SESP), saltmarsh sparrows (SALS), and Nelson's sparrows (NESP) in relation to ambient temperature. Linear mixed-effects model predictions (bold lines) are shown for each species, alongside individual trajectories (faint lines).

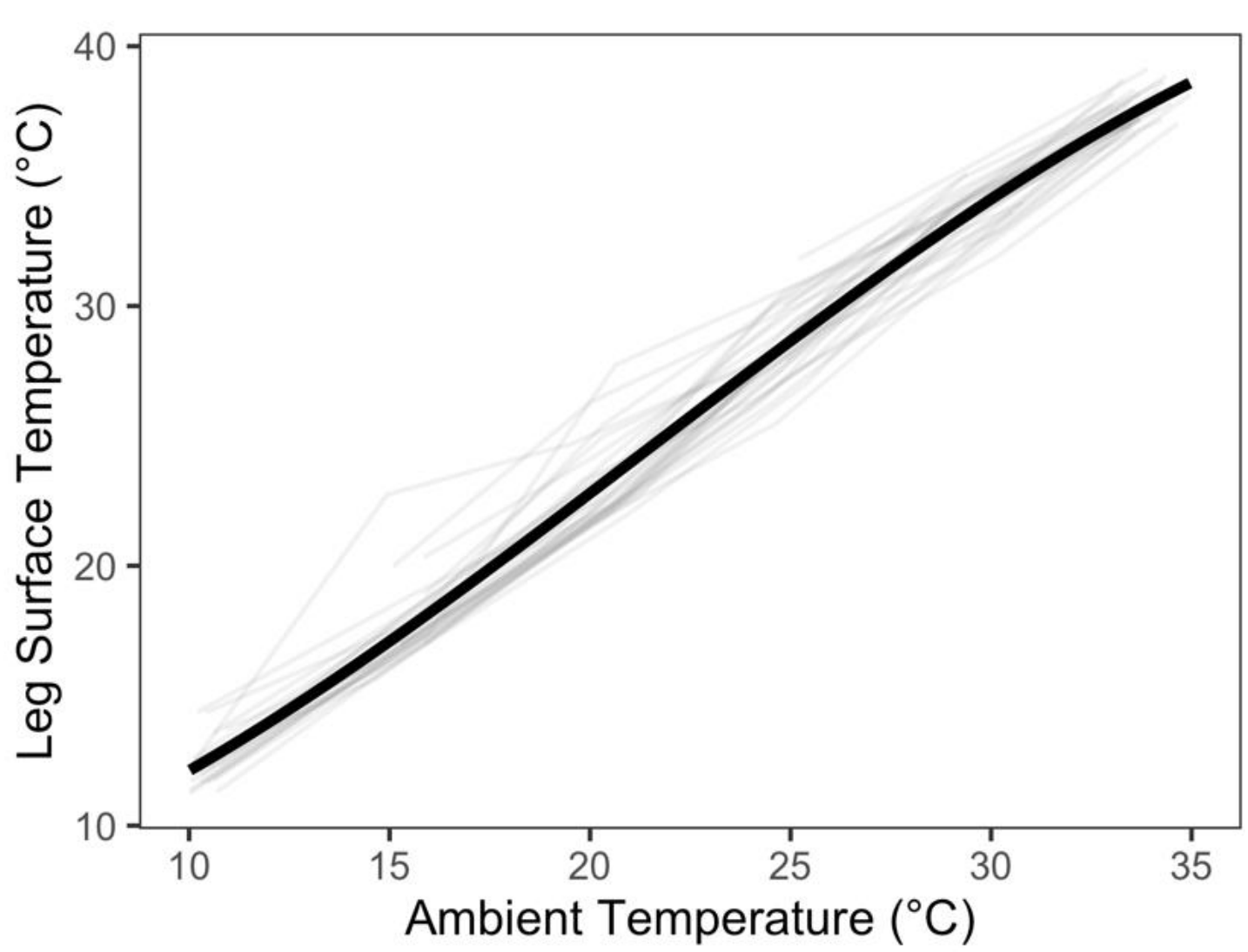

Fig. S3. Relationship between ambient temperature and leg surface temperature across all individuals. Thin gray lines show measured leg surface temperatures for each bird across ambient temperature conditions, while the solid black line represents predictions from the top performing linear mixed-effects model, which included linear, quadratic, and cubic effects of ambient temperature (Ta) and a random intercept for individual identity.

Journal of Experimental Biology • Supplementary information

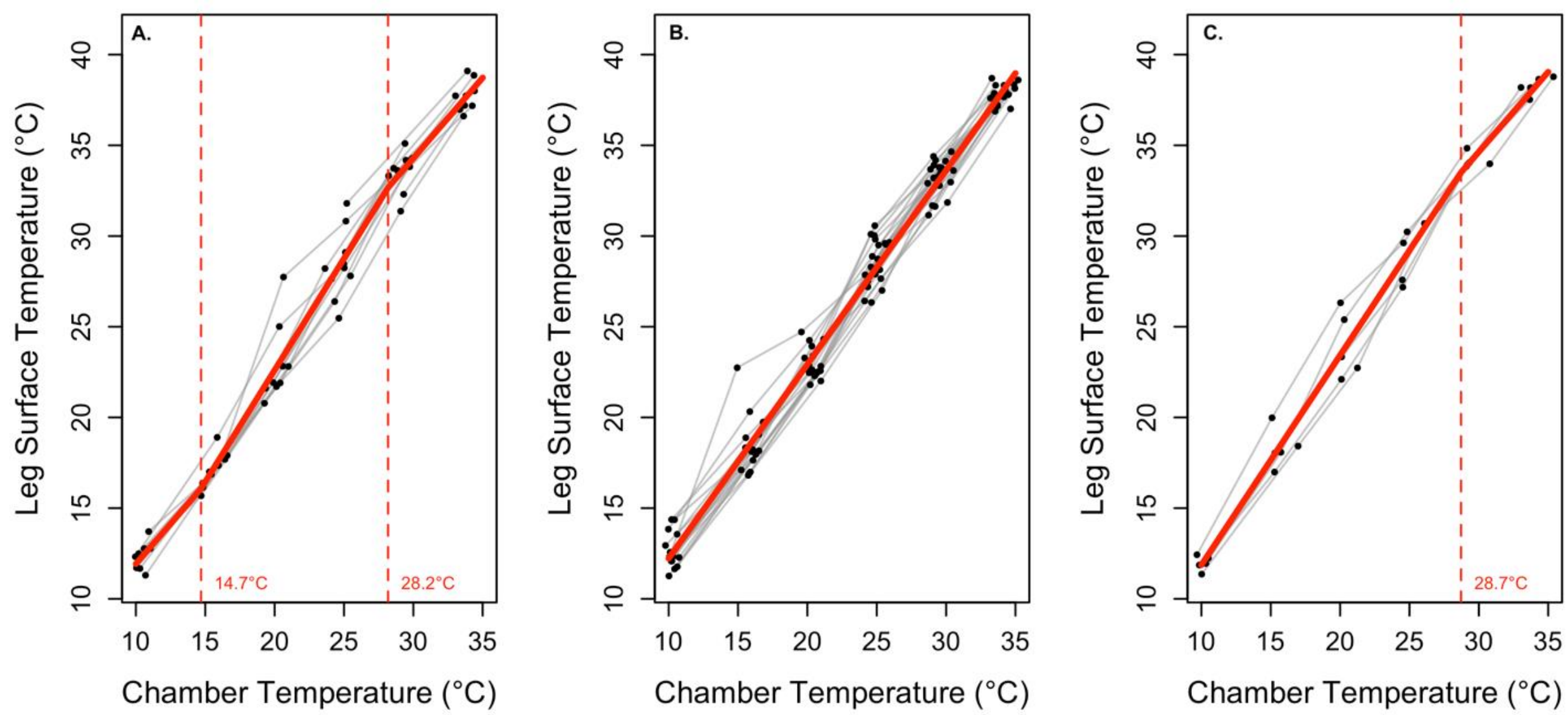

Fig. S4. Relationship between chamber temperature (°C) and leg surface temperature (°C) for seaside sparrows (A), saltmarsh sparrows (B), and Nelson’s sparrows (C). Each black dot represents an individual data point, with gray lines connecting repeated measures from the same individual. Red solid lines represent fitted relationships. For seaside sparrows, the fitted line shows the exploratory two-breakpoint segmented regression, with estimated breakpoints at 14.7°C and 28.2°C indicated by dashed vertical lines. For saltmarsh sparrows, the red line represents the linear mixed-effects model, which was better supported than the one-breakpoint segmented mixed-effects model. For Nelson's sparrows, the red line represents the one-breakpoint segmented mixed-effects model, with the estimated breakpoint at 28.7°C indicated by the dashed vertical line. Breakpoints are marked with their respective temperature values.

Journal of Experimental Biology • Supplementary information
